# Controlled Photoiniferter RAFT-Based Development of Linear Polymers Induced by the Presence of a Peptide to Produce Synthetic Antibody Substitutes Applicable to Immunofluorescence Imaging Techniques

**DOI:** 10.1021/acs.analchem.5c05598

**Published:** 2025-12-08

**Authors:** Lucía Diez-Caballero, Imanol González-Burguera, Miquel Saumell-Esnaola, Nora Unceta, M. Aránzazu Goicolea, Joan Sallés, Ramón J. Barrio, Gontzal García del Caño, Alberto Gómez-Caballero

**Affiliations:** †Departments of Analytical Chemistry, ‡Neurosciences, and §Pharmacology, Faculty of Pharmacy, 82992 University of the Basque Country UPV/EHU, 01006 Vitoria-Gasteiz, Spain; ∥ Bioaraba, MetaboloMIPs, 01008 Vitoria-Gasteiz, Spain; ⊥ Bioaraba, Neurofarmacología Celular y Molecular, 01008 Vitoria-Gasteiz, Spain

## Abstract

The fabrication of synthetic receptors that mimic the behavior of antibodies is attracting widespread attention, given their affinity and improved stability over those of their natural counterparts. In accordance with the mutually induced-fit principle, where flexible chains mutually induce the organization of each other, herein, we describe the fabrication linear copolymers as complementary chains to peptide epitopes. The production of these linear polymers is performed via controlled solid-phase polymerization by reversible addition–fragmentation chain transfer using a photo*iniferter* (PI-RAFT), pouring the monomer mixture onto glass beads having a peptide immobilized on their surface. This peptide influences the monomer arrangement in linear polymers generated at the surface, functioning as a template. PI-RAFT polymerization has enabled the production of polymers with half, equal to, or twice the size of the target peptide, the latter showing maximum affinity (*K*_D_ *:* 9.09 ± 0.27 nM). The present study focuses on the cannabinoid CB_1_ receptor as a target protein and its linear C-terminal intracellular sequence as an epitope targeted by the polymers described herein. Accordingly, a synthetic 12 amino acid peptide matching that sequence was used as a template, achieving polymers with outstanding recognition capacity toward the target protein, also being evidenced here by their applicability to dual-labeling immunofluorescence assays toward CB_1_ receptor-expressing cells.

## Graphical abstract

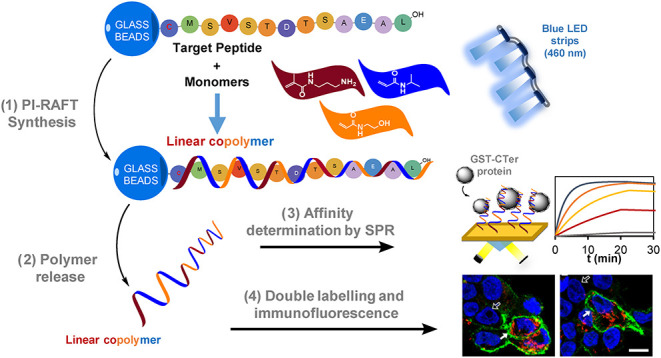

## Introduction

Inspired by Fischer’s “lock-and-key” analogy and Koschland’s induced-fit model to describe enzyme–substrate interactions, in the last decades, the biological molecular recognition event has focused a great deal of research among scientists to achieve biomimetic synthetic receptors with performances comparable to those of living systems.^1–3^ Natural receptors present excellent complementarities for target ligands, both in terms of spatial accessibility and functional groups existing in binding regions, which determines receptor specificity and biological responses triggered as a result of binding interaction.^4^ In this context, achieving this level of complementarity is often a priority pursued by researchers in the field of synthetic receptors, which in turn demands a wide knowledge and deep understanding of binding mechanisms underlying biomolecular recognition in physiologic aqueous media.^5^ Imitating the biological binding event in aqueous systems is particularly challenging, especially considering that water competes with polar interactions taking place in binding pockets, which makes binding less favorable because of the high desolvation cost.^6^ To overcome this, hybrid interaction mechanisms should be considered when designing synthetic receptors targeting macromolecules, combining hydrogen bonds alongside hydrophobic forces, electrostatic interactions, or even metal coordination bonds, replicating, to some extent, biological behavior.^7^

The design and fabrication of synthetic receptors for macromolecular targets has been conducted, in most cases, under three approaches, by affinity screening from a library of preformed synthetic polymers, using polymer-scaffolded dynamic combinatorial libraries, or by the application of molecular imprinted technology.^8–10^ Molecularly imprinted polymers (MIP) are tailor-made synthetic materials with antibody or enzyme-like binding behavior, overcoming common drawbacks of natural receptors including their lower stability under nonphysiological conditions, higher production costs, and, in some instances, poor batch-to-batch reproducibility.^11,12^ Furthermore, since MIPs are not proteins, they are resistant to proteolysis and microbial degradation. Relying on their success, research on imprinted materials is experiencing considerable evolution toward the production of MIPs for macromolecular targets, including peptides, proteins, viruses, or even whole cells,^13,14^ to produce artificial receptors that resemble more to monoclonal antibodies, as regards specificity and binding performance.^15^ Within this scenario, size reduction of imprinted materials to the nanoscale has proven to be determinant,^16,17^ and has provided MIPs with the distinctive properties of nanomaterials, improving their binding capacities and mass transfer kinetics.^18^ Accordingly, molecularly imprinted nanoparticles (MIN) exhibiting antibody or enzyme-like behavior have been successfully produced so far, and have found potential applications in the biomedical field.^19^ However, further research is still needed to produce nanoparticles of a few nanometers having homogeneous size and binding site distributions with minimal cross-reactivity to further approach or even improve binding performances that usually present their natural counterparts.

As an alternative to cross-linked MIP materials, other polymer formats have also been explored as potential synthetic receptors for proteins. Schrader’s group developed a set of flexible linear copolymers combining methacrylamide-based comonomers at different relative molar ratios. That group of monomers was selected based on the most characteristic residues on protein surfaces, being capable of interacting with acidic, polar, aromatic, and nonpolar amino acids.^20^ The statistical copolymerization of selected monomers by free radical polymerization in the absence of any template or cross-linker gave rise to flexible polymers capable of binding to protein surfaces by an induced-fit mechanism. Likewise, Shea, Miura et al. explored linear formats of synthetic receptors producing a library of multifunctional linear poly-*N*-isopropylacrylamide-based copolymers and their capability to capture the peptide melittin.^21,22^ Linear polymers produced by chemically initiated free radical polymerization have also been examined as anti-infective materials targeting pathogens.^23^ Unlike previous ones described above, these linear polymers were synthesized in the presence of a template, namely, the C-terminal 10 amino acid fraction of the PhrA peptide, a signaling peptide for the TprA receptor in *Streptococcus pneumoniae* that modulates quorum sensing (QS). This combination of imprinted materials and linear polymer formats was further explored by our research group, following Schrader’s postulates about the induced-fit mechanism between flexible linear polymers and proteins. Accordingly, a linear polymer having different functionalities to target the globular protein lactoferrin was synthesized, using an *iniferter*-based living polymerization procedure under ultraviolet (UV) light.^24^ The produced polymer was implemented as an artificial receptor on ELISA assays to determine lactoferrin in urine.

Inspired by the way in which linear polymers adapt to rigid macromolecular targets, we aimed to explore the possibility of producing linear polymers as artificial receptors that can adapt to flexible targets, such as linear peptides. This approach was based on the mutual induced-fit principle, where flexible chains mutually induce the organization of each other, resulting in strong interaction with high specificity.^25^ Polymer chains would presumably undergo multivalent binding of multiple complementary functionalities with target peptides, in a similar way that Velcro works.^26^ To reach the above goal, we considered using the reversible addition–fragmentation chain transfer (RAFT) polymerization as an efficient controlled polymerization approach, since it allows for having greater control over the polymer architecture and molecular weight.^10,27^ The RAFT process requires a continuous exogenous radical source to initiate polymerization and to compensate unavoidable irreversible termination,^28^ which has traditionally been achieved through thermal activation of conventional initiators.^29^ However, high temperatures may be counterproductive for heat-sensitive monomers or templates, and may influence the strength of template-monomer complexation, which determines the homogeneity of imprinted sites.^30^ In this context, the use of light for RAFT polymerizations attracts special interest since photo-CTAs allow performing polymerizations at room temperature. However, as conventional photoinitiators do not prevent irreversible termination in RAFT processes,^31^ other strategies emerged recently, such as photo*iniferter* (PI-RAFT) and photoinduced electron transfer (PET-RAFT) RAFT polymerizations.^32^ The former is based on direct activation of the CTA with UV or visible light, whereas the latter uses an additional photoredox catalyst.^28^ This experimental work has been focused on PI-RAFT methodology, to avoid the use add a photocatalyst that may interfere with the peptide-induced solid-phase synthesis of linear polymers. This has enabled the synthesis of fit-for-purpose linear polymers with defined molecular weights.

Herein, we report the controlled production of flexible linear polymers as artificial receptors specifically designed to target the intracellular C-terminal (C-Ter) end of the cannabinoid CB_1_ receptor. This protein was selected as a proof of concept based on our previous experience,^12,33^ and because it represents a biologically and pharmacologically relevant model for testing alternative molecular recognition strategies. Furthermore, it is one of the most abundantly expressed G protein-coupled receptors (GPCR) in the mammalian central nervous system and its intracellular region plays a key role for receptor desensitization and internalization.^34^ The CB_1_ receptor is a key modulator of many physiological functions,^35^ and research is still needed to further exploit its potential as a therapeutic target.^34^ In this light, developing and validating reliable research tools for CB_1_ detection, purification, and analysis is essential, with natural antibodies being currently the most widely used tools for this purpose. However, despite careful selection and validation, antibody-based approaches still present inherent shortcomings that compromise their reproducibility. Accordingly, developing alternative molecular recognition systems has become increasingly necessary, to address the structural complexity and signaling diversity of GPCRs, with the CB_1_ receptor standing here as a paradigmatic example.^36,37^

## Experimental Section

### Chemicals

Glass beads 150–212 μm in diameter were purchased from Merck (Spain). The silanes (3-aminopropyl)­triethoxysilane (APTES) (99%), (3-iodopropyl)­trimethoxysilane (IPTMS) (95%), and 1,2-bis­(triethoxysilyl)­ethane (BTESE) (95%), the RAFT agent 4-((((2-carboxyethyl)­thio)­carbonothioyl)­thio)-4-cyanopentanoic acid, and the monomers *N*-isopropylacrylamide (NIPAm) (97%), *N*-hydroxyethyl acrylamide (HEAA) (97%), and *N*-(3-aminopropyl) methacrylamide hydrochloride (3-APMA) (98%) were also purchased from the same company. Succinimidyl iodoacetate (SIA) (97%), disuccinimidyl glutarate (DSG) (97%), and (+)-biotin *N*-hydroxysuccinimide ester (biotin-NHS) (98%) were acquired from Fisher Scientific (Spain).

As a target peptide, a synthetic peptide of 12 amino acids having an additional cysteine was used (C-MSVSTDTSAEAL) (C-Ter), whereas the scrambled version of that sequence (LSMDEVSTATSA) was used as a control. Both the target peptide (96.03% purity, Maldi-TOF (*m*/*z*): (M + H+) 1316.23) and the scrambled version of that peptide (99.08% purity, Maldi-TOF (*m*/*z*): (M + H+) 1212.64) were custom-synthesized by Caslo ApS (Denmark).

HPLC-grade solvents such as methanol, acetonitrile, acetone, and ethanol were acquired from Scharlab (Spain), and dimethyl sulfoxide (DMSO) from Panreac (Spain). All buffer solutions were formulated with ultrapure water (resistivity of 18.2 MΩ cm), which was obtained by using Elix 20 reverse osmosis and Milli-Q water purification systems from Merck (Spain). All other reagents were of analytical grade and were used without further modification.

All instruments used in this work have been included in the SI.

### Monitoring the Progress of PI-RAFT Polymerization of Linear Polymers

Number-average molecular weight (*M*_n_) evolution during polymerization and achieved polydispersities (PDI) were determined by gel filtration chromatography coupled with a refractive index and a light scattering detector (GFC-RID/LS), while liquid chromatography with a diode array detector (HPLC-DAD) was used to measure monomer consumption. For GFC experiments, a PolySep-GFC-P 3000 (3000 × 7.8 mm^2^) column having a separation range of 250–75 kDa was used together with a PolySep-GFC-P (35 × 7.8 mm^2^) guard column (Phenomenex) working both at 30 °C. The mobile phase consisted of a 60:40 mixture of methanol and 0.25 M sodium nitrate, working at a flow rate of 0.5 mL min^–1^ and an injection volume of 50 μL. Easivial polyethyleneglicol (PEG) and polyethyleneglicol/poly­(ethylene oxide) (PEG/PEO) calibration kits were used (Agilent Technologies) for system calibration. The concentration of produced linear polymers was estimated by interpolating their peak area values in a calibration curve obtained from a series of standard solutions of PEG (4 kDa) injected in the GFC system at concentrations between 0.1 and 20 g L^–1^.

On the other hand, for monomer quantification by HPLC-DAD, a ZORBAX Eclipse XDB C-18 column (4.6 × 150 mm^2^, 5 μm) and a guard column (4.6 × 12.5 mm^2^, 5 μm) were used (Agilent Technologies). The mobile phase consisted of a mixture (95:5) of acetic acid/sodium acetate buffer (10 mM, pH 6) and acetonitrile, working at a rate of 1 mL min^–1^. The system was calibrated by injecting (20 μL) standards of each monomer at concentrations between 0.25 and 10 mg L^–1^. Based on determined monomer concentrations in polymerization solutions, monomer conversion (α) and the first-order kinetics of monomer consumption were obtained using eqs 1 and 2.

1 $$\alpha =\frac{{\left[M\right]}_{0}-{\left[M\right]}_{t}}{{\left[M\right]}_{t}}\times 100$$

2 $$\ln\left({\left[M\right]}_{t}\right)=\ln\left({\left[M\right]}_{0}\right)-\mathrm{kt}$$

where [ *M* ]_0_ and [ *M* ]*_t_* are the initial concentration and the concentration at time *t* of monomer *M* , respectively, and *k* is the pseudo-first-order rate constant^38,39^ or apparent propagation rate constant (*K*_p_^app^).^40^

### Solid-Phase Synthesis of Linear Polymers

Based on the amino acids of the target peptide (MSVSTDTSAEAL) (C-Ter), the monomer composition in the polymerization mixture included 41.67% of hydrophobic monomers, like NIPAm (0.729 g, 6.25 mmol), 41.67% of hydrophilic monomers, such as HEAA (0.742 g, 6.25 mmol), and 16.67% of basic monomers, such as 3-APMA (0.456 g, 2.5 mmol). These monomers were added to a deoxygenated (30 min, N_2_) phosphate buffer solution (25 mL, 0.05 M), containing 20 g of glass beads (GB) with the C-Ter peptide attached. GB functionalization and peptide immobilization on their surface are described in SI. Next, the RAFT agent (0.019 g, 0.06 mmol) predissolved in acetonitrile (1 mL) was added, and the mixture was deoxygenated for another 10 min. Finally, the mixture was placed in the homemade photoreactor, and it was irradiated with visible blue light-emitting LED strips (460 nm), while being gently rocker-shaked.

After polymerization, the whole content of the flask was transferred into a 60 mL filtration cartridge with a polyethylene frit (20 μm) (Merck). Subsequently, the polymerization solution containing unreacted compounds and polymers not created on the GB surface were allowed to percolate through the cartridge and eventually discarded. The GB that compose the cartridge bed were washed using cold (<8 °C) ultrapure water (4 × 25 mL washes) to remove weakly bound polymers and remaining monomers. At this point, while being still bound to template of the solid phase, linear polymers were biotinylated, as an additional step. To this end, phosphate buffer (25 mL) containing biotin-NHS (5 mg) was added to the cartridge and left to react for 2h. Following this, 4 × 25 mL washes with cold (<8 °C) ultrapure water were done to remove unreacted biotin. Biotinylation was done only for polymers that were to be used in immunofluorescence experiments as antibody substitutes. Finally, the cartridge was preheated to 65 °C in a water bath, and linear polymers were eluted from the GB surface percolating an ethanol:NaCl (0.25 M) mixture (1:1) at 65 °C (4 × 25 mL). Those 100 mL samples were concentrated down to ∼20 mL using a rotatory evaporator. Finally, NaCl was removed from the solution using Supelclean C18 SPE cartridges (2 g of bed weight) (Merck), recovering the linear polymers in 5 mL of methanol (5 mL), which was evaporated to almost dryness and redissolved in ultrapure water (1 mL).

### Preparation of Gold Sensors for Surface Plasmon Resonance Experiments

Gold sensors used for SPR experiments were activated by adding acidic piranha (400 μL, 3:1 H_2_SO_4_/H_2_O_2_) to the gold surface at 30–35 °C for 3 min. Thereafter, the sensor was washed with water, dried with nitrogen, and treated with plasma in an HPT-100 air-plasma system (Henniker) for 5 min. Subsequently, the sensor was immersed for 24 h in an ethanol:water mixture (95:5) containing 5% of APTES and acidified with acetic acid (1%). Prior to sensor immersion, the mixture was preheated to 70 °C. After silanization, the sensor was gently rinsed with water and acetone, and dried in an oven (120 °C, 1h). Finally, it was installed in the SPR instrument for attachment of either the target C-Ter peptide or the synthesized linear polymers. Immobilization of these ligands was done only on the gold sensor surface of the primary channel (ch.1), while the secondary channel (ch. 2) was used as reference, which was treated using the same reagents as ch.1 except for the ligand.

To immobilize the C-Ter peptide, the sensor was preconditioned injecting a NaOH (10 mM) solution containing 2 M NaCl, and afterward, SIA was injected (0.03 M) predissolved in mixture of phosphate buffer (0.025 M, pH 7.4) and DMSO (50:50). Next, four consecutive injections of a solution of the C-Ter peptide (3 mg mL^–1^) in borate buffer (0.1 M, pH 8.3) were made, to maximize peptide attachment. Finally, unreacted iodines were blocked by injecting mercaptoethanol (0.1 M) in borate buffer. All of these injections were run through both ch.1 and 2, except for the injection of the peptide, made only on ch.1.

Apart from the above, linear polymers were also immobilized on gold sensors silanized with APTES, as described above. To this end, a solution of DSG (400 μL, 0.03 M) in dry DMSO was deposited over the entire sensor surface, and it was left to react for 1h. After that, the sensor was installed in the SPR, and immediately a solution of the linear polymer (10^–5^ M) in phosphate buffer (0.1 M, pH 8) was injected through ch.1. Unreacted succinimidyl groups were blocked by injecting ethanolamine (0.5 M, pH 8) through both channels.

Both sensor modification approaches were performed using phosphate buffer (0.1 M, pH 7.4) containing NaCl (0.15 M) and Tween 20 (0.05%) (PBS-T) as running buffer, and all mentioned injections were made at a flow rate of 20 μL min^–1^.

### Surface Plasmon Resonance Experiments

The gold sensor, having the C-Ter peptide attached, was installed first in the SPR to examine the binding affinities of linear polymers of different lengths. Trizma (0.1 M, pH 7.4) was selected as running buffer, 10 μL min^–1^ as flow rate, and 37 °C as temperature. Under these conditions, linear polymers with *M*_n_ of 964.63 g mol^–1^, 1621.85 g mol^–1^, and 2936.29 g mol^–1^ were injected in the SPR, which were half (0.5×), equal to (1×), or twice (2×) the length of the C-Ter, at concentrations between 10 nM and 2.5 μM.

Second, a gold sensor having the 2× linear polymer immobilized was used to determine the binding affinity of the polymer and the recombinant glutathione S-transferase tagged fusion protein GST-CB1_414–472_ (GST-CTer), produced in our lab as described elsewhere.^33^ Protein concentrations between 10 nM and 100 nM were injected into the system at 10 μL min^–1^, working under the same conditions described above. Next, a truncated version of the fusion protein (GST-CB1_414–442_) was injected as a control, at concentrations ranging from 100 nM to 1 μM. The control protein was identical to the GST-CTer fusion protein but truncated at amino acid 443 (GST-Δ443), being devoid of the last 30 amino acids, and, therefore, not carrying the target C-Ter epitope sequence. Sensor regeneration between measurements was performed using 0.1 M glycine, including NaCl (50 mM) and Tween 20 (0.1%), adjusted to pH 2.5 using HCl.

All SPR data were examined using TraceDrawer analysis software (Bionavis). For kinetics evaluation, sensograms were fitted to the one-to-one binding model available with the software.

### Cell Culture Transfection and Immunofluorescence Labeling

Human embryonic kidney 293T (HEK293T) cells (ATCC) were cultured in 75 cm^2^ flasks (Corning) using DMEM (ATCC) supplemented with 10% fetal bovine serum (Merck) and antibiotics (100 U/mL penicillin and 100 μg/mL streptomycin; Gibco, Life Technologies). Once they reached ∼70–80% confluence, cells were detached with trypsin–EDTA (Gibco) and seeded onto poly-d-lysine-coated glass coverslips placed in 6-well plates. Upon reaching 70–80% confluence again, cells were transfected with 2 μg plasmid DNA per well using the pCDNA3.0 vector encoding the human CB_1_ cannabinoid receptor (pCDNA-CB_1_), using Lipofectamine 3000 (Invitrogen). After 48 h, the cells were processed for live-cell immunolabeling followed by single or dual immunofluorescence staining.

For total CB_1_ receptor immunolabeling, cells were fixed with paraformaldehyde (4%) in PBS for 5 min at 22–25 °C, washed in PBS containing gelatin (0.22%) (wash buffer), and permeabilized and blocked for 1 h at room temperature using blocking buffer (wash buffer supplemented with 0.066% saponin, 1% BSA and 1% normal donkey serum). Primary antibody and linear polymer incubation was carried out for 1 h at 37 °C by combining the anti-CB_1_ rabbit polyclonal antibody H150 (Santa Cruz Biotechnology; 1 μg/mL) and the biotinylated 2× linear polymer (10^–7^ or 10^–9^ M), both diluted in blocking buffer. After three washes with wash buffer, cells were incubated for 1 h with fluorescent secondary reagents Alexa Fluor 488-conjugated donkey antirabbit antibody (Invitrogen; 1:400) and DyLight 549-conjugated streptavidin (Vector Laboratories; 1:400) in blocking buffer. For surface CB_1_ labeling, coverslips were transferred to ice-cold Opti-MEM I (Gibco) and placed on crushed ice for 5 min to minimize receptor internalization. Cells were then incubated for 1 h at 18 °C with anti-CB1 H150 antibody (1 μg/mL in Opti-MEM I), washed three times with ice-cold wash buffer, and fixed with 4% paraformaldehyde as described above. Cells were subsequently permeabilized and blocked as above and incubated for 1 h at 37 °C with the biotinylated 2× linear polymer (10^–7^ or 10^–9^ M) in blocking buffer, followed by incubation with fluorochrome-conjugated secondary reagents as described above. Nuclei were stained with Hoechst 33342 (Merck) at a final concentration of 0.1 μg/mL in wash buffer, for 10 min at 22–25 °C. Subsequently, coverslips were rinsed twice with PBS (10 min each, at 22–25 °C) and mounted on glass slides using a homemade Mowiol-based mounting medium (Calbiochem) containing the antifade agent 1,4-phenylene-diamine dihydrochloride (Merck).

## Results and Discussion

### Tuning the PI-RAFT Methodology to Produce Fit-for-Purpose Linear Polymers

The production of linear polymers as artificial receptor systems that function as synthetic chains showing complementarity for a peptide epitope of a given protein was pursued here. To accomplish this challenge, the cannabinoid CB_1_ receptor was selected as target protein, as a proof of concept. It was intended to achieve a methodology for fit-for-purpose production of linear polymers for selected epitopes, applicable not only for the single target described in this article but also adaptable to other protein epitopes.

Epitope selection was based on three main criteria: (i) its intrinsically disordered nature, (ii) the uniqueness of this region, showing no significant homology with other proteins according to BLASTp (https://blast.ncbi.nlm.nih.gov/), and (iii) previous evidence that peptides encompassing the last 13,^41^ 15,^42^ or 31 amino acids of CB_1_,^36,37^ have served as effective antigens for generating highly selective antibodies. Indeed, the C-terminal region of the CB_1_ receptor follows the canonical architecture of class-A GPCRs, where the cytosolic tail acts as an intrinsically disordered region (IDR) conferring conformational adaptability for post-translational regulation and protein binding.^43^ Within this region, helices H8 (401–412) and H9 (440–461) adopt α-helical structure, whereas the distal segment remains flexible and unstructured in solution,^44^ as also supported by AlphaFold low-confidence scores (pLDDT < 50) typical of IDRs.^45^ Guided by this structural context, the selected 12-mer peptide (461-MSVSTDTSAEAL-472) corresponds to a solvent-exposed flexible epitope (Figure S1).

This 12 amino acid sequence was used to induce polymer formation via the solid-phase synthesis approach, instead of being free in the solution for polymerization, it was covalently immobilized on GB, and the monomers that interacted noncovalently with the peptide on the solid-phase were polymerized by PI-RAFT, discarding the polymers formed within the solution. It was intended to produce linear polymers with sizes comparable to the C-Ter, as synthetic and complementary linear chains having a monomer arrangement presumably influenced by the peptide attached to the solid-phase.

Monomer selection for producing such linear polymers was based on the amino acid sequence of the target peptide, which includes five residues (41.67%) with hydrophobic side chains—methionine (M), valine (V), 2× alanine (A), and leucine (L), five with polar uncharged side chains (41.67%)—3× serine (S) and 2× threonine (T), and two an acidic group (16.67%)—aspartic acid (D) and glutamic acid (E). Considering all of this, functional monomers capable of interacting with these amino acids were selected, such as NIPAm to target amino acids with hydrophobic side chains, HEAA for polar uncharged side chains, and 3-APMA for amino acids with acidic side chains. The linear NIPAm/HEAA/3-APMA copolymers obtained by PI-RAFT are expected to behave as flexible random coils in aqueous buffer, lacking a defined secondary structure. Their hydrophilic and ionizable composition prevents collapse or ordered folding, consistent with the behavior of (meth)­acrylamide copolymers. This polarity complements the acidic and polar nature of the target peptide, favoring ionic and hydrogen-bonding interactions and supporting a flexible-to-flexible recognition model similar to that of antibodies recognizing dynamic or partially exposed epitopes.^46^

Concerning the RAFT agent, the water-soluble compound 4-((((2-carboxyethyl)­thio)­carbonothioyl)­thio)-4-cyanopentanoic acid (CCCA) was selected, which is a yellow trithiocarbonate with maximum visible light absorption around 440 nm. Consequently, for PI-RAFT, commercial LED light strips (5 m-long 300 LED strip, 12 V, 40 W) that emit visible blue light at 460 nm were arranged around 1 L beakers and covered with aluminum foil to prepare homemade photoreactors (Figure S2).

Before the production of linear polymers complementary to the selected epitope, a thorough study of the PI-RAFT process was necessary. We aimed to determine the influence of different variables, such as monomer concentration, irradiation time, or the monomer:RAFT ratio, on monomer conversion and the number-average molecular weight (*M*_n_) of produced polymers. All of these experiments were conducted in the absence of any peptide, with the mixture containing only monomers and the RAFT agent. Initial experiments focused on determining the total monomer concentration required to produce a high yield of linear polymers with a low polydispersity. In this context, polymerizations were carried out in 25 mL of phosphate buffer (0.05 M, pH 7.4) with monomer concentrations between 0.15 and 1.2 M, consisting of 41.67% of NIPAm, 16.67% of 3-APMA, and 41.67% of HEAA. The obtained results revealed that concentrations of 0.3 M and 0.15 M showed little (<30%) or no conversion (Figure 1A), while for 0.6 M and 1.2 M, conversions reached approximately 80% after 24 h, also finding that polymerizations followed pseudo-first-order kinetics, suggesting a constant concentration of propagating radical species.^40^ Furthermore, total monomer concentrations of 0.6 M and 1.2 M (Figure S3a,b), unlike 0.3 M (Figure S3c), resulted in linear *M*_n_ evolution with respect to monomer conversion, with polydispersities ranging from 1.2 to 1.5, which is in agreement with a controlled polymerization process.^47,48^ Given that 1.2 M showed no apparent improvement over 0.6 M, the latter was selected for further experiments, as it exhibited a slightly higher apparent propagation rate (*K*_p_^app^) of 0.073 h^–1^.

**Figure 1:**
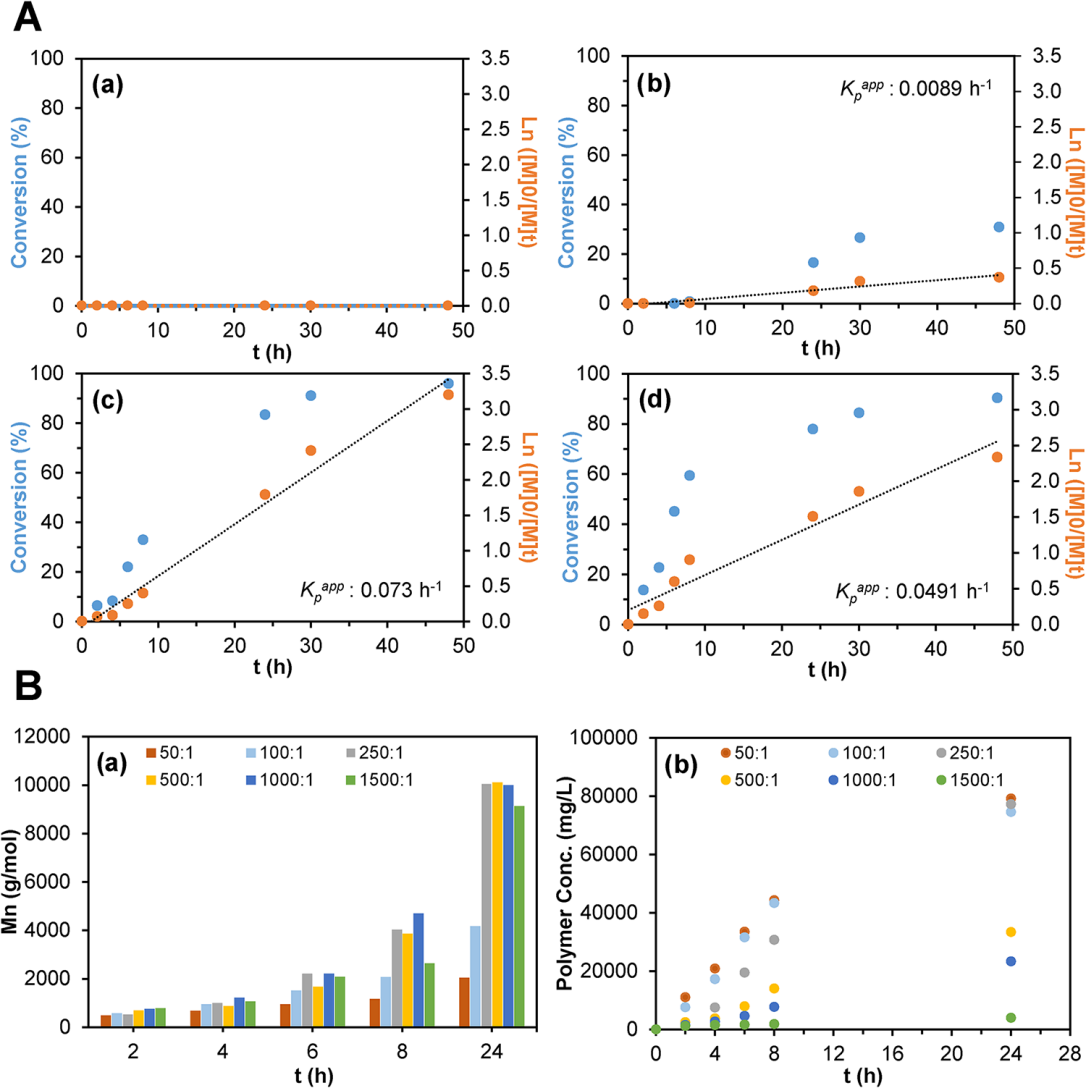
(A) Monomer conversion (%) and first-order kinetic behavior for polymerization mixtures having a total monomer concentration of (a) 0.15, (b) 0.3, (c) 0.6, and 1.2 M. (B) Overtime evolution of (a) *M*_n_ and (b) concentration, for linear polymers synthesized using different monomer:RAFT ratios. K_p_^app^: apparent propagation rate.

In addition to the above, the monomer:RAFT molar ratio also plays a key role in *M*_n_, since higher ratios contribute to fewer polymer chains in number but longer in size. In this context, ratios ranging from 50:1 to 1500:1 were examined for polymer synthesis, using a constant monomer concentration of 0.6 M. As depicted in Figure 1Ba, polymers produced at 50:1 and 100:1 were much shorter than those obtained at higher ratios, which reached approximately 10,000 g mol^–1^. Furthermore, after 24 h, solutions prepared with ratios of 1000:1 or 1500:1 presented a gel-like appearance, which interfered with proper molecular weight evolution of the polymer in solution; therefore, they were discarded. The monomer/RAFT molar ratio had also influenced the concentration of the resulting polymers (Figure 1Bb). Ratios ranging from 50:1 to 250:1 provided good polymerization yield, while for 500:1 or above, the amount of polymer was reduced to less than a half. These findings were consistent with the results depicted in Figure S4, which reveals that monomer conversion after 24 h decreased considerably from 80%, at a ratio of 250:1, to approximately 50% and 20% at 500:1 and 1500:1 ratios, respectively. In the same vein, for 500:1 or above, *K*_p_^app^ experienced a notable decrease (Figure S4), the *M*_n_ increase with monomer conversion did not follow a linear trend (Figure S5), and PD values were considerably higher, which denoted a loss of control over polymerization. Based on these results, we considered 250:1 as the most appropriate monomer/RAFT molar ratio to accomplish the intended objectives, achieving polymers of ∼10 kDa after 24 h, without observing any loss of livingness.

### Adaptation of the PI-RAFT Methodology to Solid-Phase Synthesis of Linear Polymers Induced by the C-Ter Peptide

After tuning the PI-RAFT procedure to the purpose of this work, we explored whether polymer evolution under detailed PI-RAFT conditions was altered by the presence of glass beads (GB) or not, as polymer synthesis was later conducted in the presence of GB having the C-Ter peptide on the surface. Given that the PI-RAFT process was photochemically controlled, it was crucial to determine if the addition of glass microparticles to the reaction mixture reduces light penetration, giving rise to shorter polymers. Results concerning these experiments are discussed in SI (Figure S6), which revealed that GB amounts not exceeding 20 g may be recommended. Additionally, covalent immobilization of the target peptide on GB was also explored by using two approaches. One of them focused on GB silanization with (3-aminopropyl)­triethoxysilane (APTES), and the other with (3-iodopropyl)­trimethoxysilane (IPTMS) (Figure S7). As outlined in the SI, the APTES protocol was chosen here, as it provided a higher yield of grafted peptide and because residual iodines introduced by IPTMS interfered with the RAFT process (Figure S8).

Following the above, PI-RAFT mediated solid-phase synthesis of linear polymers targeting the C-Ter sequence of the CB1 receptor was conducted, carefully considering the length of the linear polymers to be produced to maximize the binding affinity for the target sequence. Accordingly, we hypothesized that polymers with molecular weights of half (0.5×), equal to (1×), or twice (2×) the molecular weight of the target peptide may be explored as possible candidates since longer chains may contribute more to nonspecific binding. Given that the molecular weight of the target peptide was 1314.44 g mol-1, the polymerization strategy was designed to produce linear polymers with *M*_n_ of 964.63 g mol^–1^, 1621.85 g mol^–1^, and 2936.29 g mol^–1^, corresponding to 0.5×, 1×, and 2× polymers, also considering the presence of the RAFT agent (307.41 g mol^–1^). Figure 2A displays a scheme illustrating the pursued idea, showing structures and number-average molecular weights that may be desirable in a hypothetical ideal situation. To synthesize simultaneously the three polymer variants, three homemade photoreactors were assembled, which were independently calibrated to determine the linear dependence of *M*_n_ on LED irradiation time (Figure S9).

**Figure 2:**
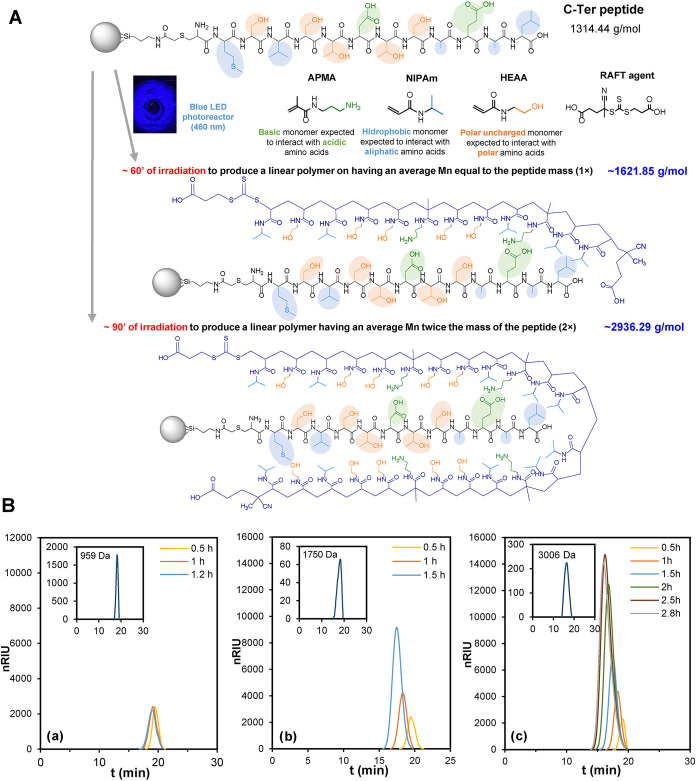
(A) Schematic representation of the solid-phase synthesis 1× and 2× linear polymers on the surface of glass beads having the target C-Ter peptide attached. The structures and number-average molecular weights depicted are those that would be desired in an ideal situation. (B) Evolution of chromatographic peaks extracted from complete GFC-RI/LS chromatograms corresponding linear polymers formed in solution at shown irradiation times (main graph), and on the solid phase (inset) after polymerization is complete. (a) Linear polymers (0.5×) synthesized in photoreactor 3, (b) linear polymers (1×) synthesized in photoreactor 2, and (c) linear polymers (2×) synthesized in photoreactor 1. The insets show *M*_n_ data corresponding to *n* = 3 batches of linear polymers.

Once all conditions were established, peptide-induced solid-phase syntheses of three batches of 0.5×, 1×, and 2× linear polymers were produced (Figure 2B), using the corresponding irradiation times. Since polymerizations were conducted in a mixture containing GB, polymers may have formed both on the solid phase and in solutions. Therefore, during polymerization, small aliquots were extracted from the reaction mixture and analyzed by GFC to monitor the *M*_n_ evolution. Upon completion of polymerization, the GB coming from the three batches were combined and washed, and the linear polymers remaining bound to the peptide on the GB surface were released. Linear polymers were analyzed by GFC obtaining the peaks illustrated in Figure 2B. Regarding linear polymers formed in solution, the observed *M*_n_ value for 2× polymers was 2888 ± 188 g mol^–1^ (*n* = 3), for 1× polymers 1735 ± 89 g mol^–1^ (*n* = 3), and for 0.5× polymers 841 ± 41 g mol^–1^, being very close to linear polymers generated on the solid-phase (Figure 2B, inset). The concentrations of such linear polymers generated on the surface, estimated by GFC-RID/LS, were 4.35 × 10^–5^, 1.57 × 10^–5^, and 6.565 × 10^–5^ mol L^–1^ for 2×, 1×, and 0.5× polymers, respectively.

^1^H NMR analyses (Figure S10) carried out for the linear polymers produced in the presence of the peptide revealed an estimated monomer composition of 33% NIPAm, 44.3% HEAA, 17.8% 3-APMA, and 4.9% RAFT agent (Figure S11). This composition was considerably different from control polymers synthesized in the absence of the peptide (Figure S11), whose estimated monomer composition was 43.7% NIPAm, 34.2% HEAA, 18.0% 3-APMA, and 4.1% RAFT agent. Based on all these data included in the SI, we can conclude that the linear polymers created on the solid phase and those statistically generated in solution present different monomer abundances, even though their *M*_n_ values are comparable. The linear polymer created in the presence of the peptide shows a higher amount of the polar monomer HEAA alongside a lower amount of the hydrophobic monomer NIPAm. This may be attributed to steric hindrance in NIPAm monomers caused by the isopropyl group, which may prevent the monomer from proper noncovalent interaction with the peptide surface. Thus, the monomer is less likely to be incorporated into the polymer chain. Conversely, the higher hydrophilicity of HEAA would favor its dissolution in the aqueous polymerization medium, and therefore, its diffusion to the peptide proximities. This would be beneficial for establishing peptide-HEAA interactions, which will promote the presence of this monomer in the polymer chains created around the peptide attached to the solid phase. Considering the average *M*_n_ obtained for 2× polymers (Figure 2c), and the estimated composition by NMR, the target linear polymer may consist of approximately ∼8 NIPAm, ∼10 HEAA, and ∼4 3-APMA units.

### Binding Experiments Conducted by SPR Using Linear Polymers as Ligands Immobilized on Gold Sensor Slides

The binding behavior of polymers of different sizes was examined by multiparametric surface plasmon resonance (MP-SPR). First, it was intended to determine how the length of the synthesized linear polymers influenced the binding affinity for the C-Ter. To this end, experimental conditions that could influence such binding, including temperature, NaCl concentration, and injection flow rate, were initially optimized to reduce nonspecific signal (Figure S12). These results are discussed in the SI. Next experiments focused on injecting increasing concentrations of 0.5×, 1×, and 2× polymers in the SPR system to assess their binding behavior for the C-Ter peptide (Figure S13). The binding of 0.5× polymers was minimal, with specific SPR binding signals almost undetectable; therefore, this polymer was discarded for further experiments. Between 1× and 2× polymers, the latter was showing higher affinity for the C-Ter peptide (*K*_D_: 9.09 ± 0.27 nM) (Table 1); therefore, this polymer was selected as a better ligand. These results are discussed further in the SI.

**Table 1:** Summary of Binding Parameters Determined by MP-SPR after Curve Fitting to the Langmuir One-Site Binding Model

analyte	C-Ter peptide		GST-CTer protein
ligand	linear polymer ×2	linear polymer ×1	linear polymer ×2
*K*_D_ (nM)	9.09 ± 0.27	181 ± 15	11.90 ± 0.01
*k*_d_ (s^‑1^)	(5.20 ± 0.08) × 10^–5^	(21.2 ± 0.1) × 10^–5^	(5.760 ± 0.002) × 10^–5^
*k*_a_ (M^‑1^ s^‑1^)	(5.72 ± 0.08) × 10^3^	(1.17 ± 0.09) × 10^3^	(4.830 ± 0.002) × 10^3^
*B*_max_ (Deg)	0.02	0.02	0.02
*U*-value (%)	11	10.5	10.5

2× linear polymers were examined next to determine their binding affinity for a GST-tagged fusion protein encompassing the cytosolic domain of the native human CB_1_ cannabinoid receptor (GST-CTer), spanning residues 414 to 472 and thus including the target 12 amino acid segment (amino acids 461–472) used as template for polymer synthesis. To this end, the 2× polymer was immobilized on the surface of a gold sensor. Figure 3 depicts Δθ signals relative to GST-CTer binding to ch.1 before (Figure 3Ba) and after subtraction of reference signal from ch.2 (Figure 3Bb). Specific GST-CTer binding to the linear polymer was concentration-dependent (Figure 3Bb), showing a *K*_D_ of 11.90 ± 0.01 nM (Table 1) after fitting the curves to the one-site binding model. For comparison, we used a truncated version of the GST-CTer protein (GST-Δ443), lacking the final 30 amino acids of the cytosolic C-terminal tail and, therefore, not containing the target C-Ter sequence (Figure 3A).

**Figure 3:**
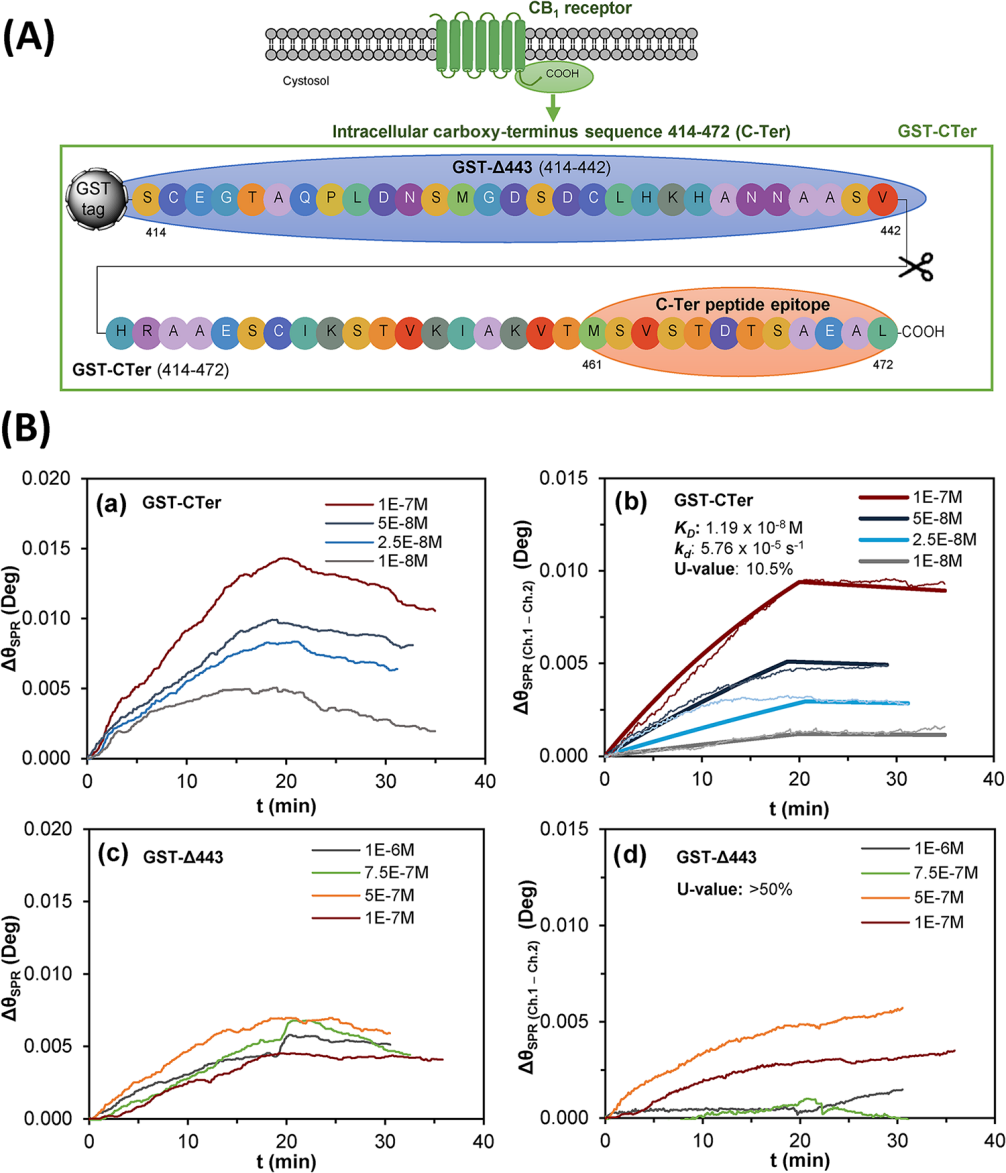
(A) Schematic representation of the amino acid sequences of the recombinant GST-CTer and the truncated GST-Δ443 fusion proteins. (B) Recorded SPR sensograms during the injection of increasing concentrations of the GST-CTer (a, b) or the GST-Δ443 (c, d) proteins. Sensograms (a, c) correspond to total binding in channel 1 for GST-CTer and GST-Δ443, respectively, whereas (b, d) depict ch.1-to-ch.2 signal difference, showing specific binding. A gold sensor with 2× linear polymers as ligands was used in all these experiments.

Figure 3Bd depicts SPR sensograms for GST-Δ443 after reference signal subtraction, showing considerably lower Δθ signals than those observed for GST-CTer, even for 10^–6^ M, which exceeds 10-fold the highest injected concentration tested for GST-CTer. Furthermore, little differences were observed across injected GST-Δ443 concentrations (Figure 3B (c, d)), and no clear signal-to-concentration dependence was appreciated. Actually, the obtained sensograms did not fit to the Langmuir one-site model, showing a uniqueness value (*U*-value) higher than 50%. *U*-values exceeding the acceptable 15% threshold indicate that kinetic parameters such as *k*_a_, *k*_d_, or *B*_max_ are correlated; therefore, the absolute values of these parameters cannot be accurately determined.^49^ Therefore, it may be concluded that removing the C-terminal sequence containing the molecular target negatively affected the specific recognition by the linear polymer.

### Competition Experiments

To explore in more depth the specific binding event between the 2× linear polymer and the target GST-CTer protein, competition experiments were conducted using as competitors either the C-Ter peptide or its scrambled version. In each case, a solution containing 1, 5, or 10 nM peptide was first injected into the SPR instrument having a gold sensor with the 2× linear polymer, with the purpose of blocking binding sites capable of recognizing the target C-Ter sequence. Immediately after, a 100 nM solution of the GST-CTer protein was injected, containing 1, 5, or 10 nM of either the C-Ter or the scrambled peptide to prevent dissociation, and angle shift (Δθ) signals induced by protein binding were monitored.

4a, b, and c depict recorded Δθ during C-Ter peptide injection (blue-shaded section), followed by the coinjection of the GST-CTer protein and the C-Ter peptide (green-shaded section). A gradual transition from Figure 4a–c illustrates two concurrent effects: first, a progressive rise of the recorded signal for the C-Ter (blue section), as peptide concentration increases from 1 to 10 nM, reflecting the occupation of recognition sites prior to GST-CTer injection; and second, a progressive reduction of the channel 1-to-2 signal difference during GST-CTer coinjection with the peptide (green section). Thus, 1 nM of peptide caused only partial blocking of sites, as a substantial channel 1-to-2 signal gap remained upon GST–CTer injection (Figure 4a). At 5 nM (Figure 4b), this difference narrowed, whereas at the highest tested C-Ter concentration (10 nM), the response in the reference channel exceeded that observed in the primary channel, which was attributable to a complete loss of specific binding, suggesting nonspecific interactions with the sensor surface in both channels. To clarify these observations, the channel 1-to-2 signal difference is represented in Figure 4d–f. As it can be observed, GST-CTer signal increments are lower as the concentration of the competitor increases, not observing any signal increment attributable to the protein if 10 nM of peptide were injected first (Figure 4f, green section). In fact, the 1-to-2 difference in channel 2 tends to decline, as a result of the higher signal in channel 2 compared to channel 1.

**Figure 4:**
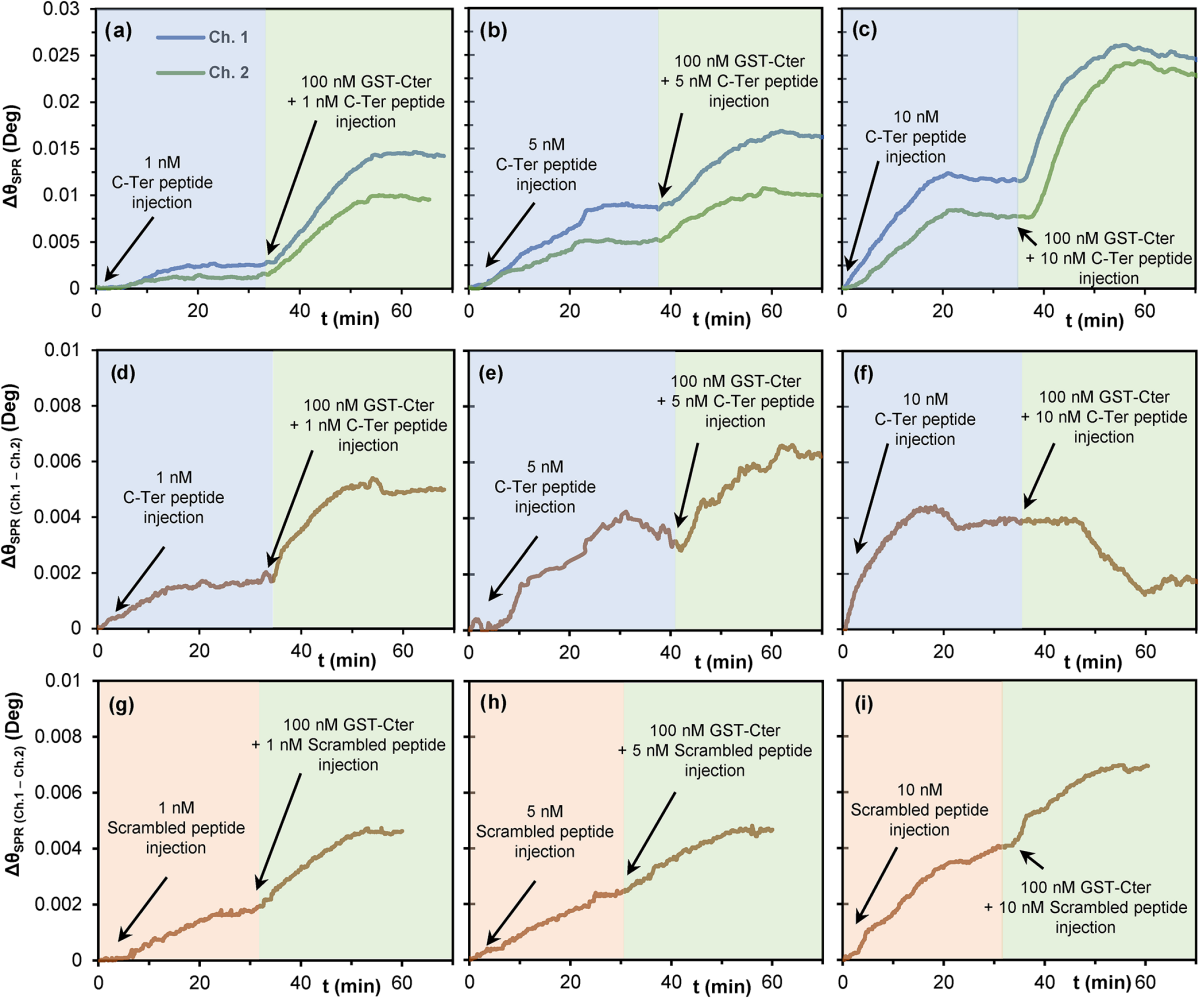
SPR sensograms recorded in the primary (ch.1) and reference (ch.2) channels during the injection of (a) 1 nM, (b) 5 nM, and (c) 10 nM of the C-Ter peptide followed by 100 nM of the GST-CTer protein. (d, e, f) Sensograms depicting ch.1-to-ch.2 signal difference for sensograms a-c. Similarly, g-i correspond to ch.1-to-ch.2 signal differences after injecting (g) 1 nM, (h) 5 nM, and (h) 10 nM of the scrambled peptide followed by the GST-CTer protein.

Based on these results, it was concluded that the C-Ter peptide competes with the GST-CTer protein for binding to the polymer. However, it remained to be determined whether the scrambled version of the peptide could also work as a competitor or not. This would provide further insight into whether binding occurs preferentially at the 12 amino acid peptide sequence selected as the epitope. At the highest scrambled peptide concentration (10 nM), the signal relative to GST-CTer binding remained appreciable (green-shaded section in Figure 4i), suggesting that the scrambled peptide does not bind to the linear polymer to the same extent as the target C-Ter peptide. The relative reduction of Δθ upon GST-CTer coinjection after preblocking with the target or scrambled peptide is depicted in Figure S14. Injection of 5 nM of the target peptide reduced the GST-CTer signal by approximately 50%, while 10 nM completely abolished it. Conversely, the scrambled peptide produced only a modest decrease of around 30%.

### Application of Synthetic Linear Polymers as Artificial Antibodies for CB_1_ Receptor Detection in Double Immunofluorescence Labeling

Given the high-affinity binding profile of the linear polymers, with an equilibrium dissociation constant (*K*_D_) in the low nanomolar range (∼10 nM), comparable to values reported for natural antibodies used in similar applications, we next sought to assess their performance in a dual immunofluorescence labeling approach on HEK293T cells transiently transfected with the human CB_1_ receptor. For this purpose, we selected concentrations of 10^–7^  M and 10^–8^ M for the polymer, within the range typically used for anti-CB_1_ primary antibodies in immunocytochemical protocols,^37^ in combination with a previously validated rabbit polyclonal antibody raised against the extracellular N-terminal region of the human CB_1_ receptor,^36^ allowing parallel assessment of target recognition at distinct receptor domains. To evaluate polymer recognition specificity, CB_1_ receptor-transfected HEK293T cells were colabeled with the N-terminal antibody and the linear polymer and visualized using Alexa Fluor 488-conjugated donkey antirabbit antibody and Alexa Fluor 549-conjugated streptavidin secondary reagents, respectively. Two labeling conditions were applied. On the one hand, cells were fixed and permeabilized prior to the simultaneous addition of antibody and polymer, enabling detection of both surface and intracellular epitopes (Figure 5A–F). On the other hand, antibody binding was restricted to the extracellular domain by first applying it to live cells, followed by fixation and subsequent incubation with the polymer (Figure 5G–L).

**Figure 5:**
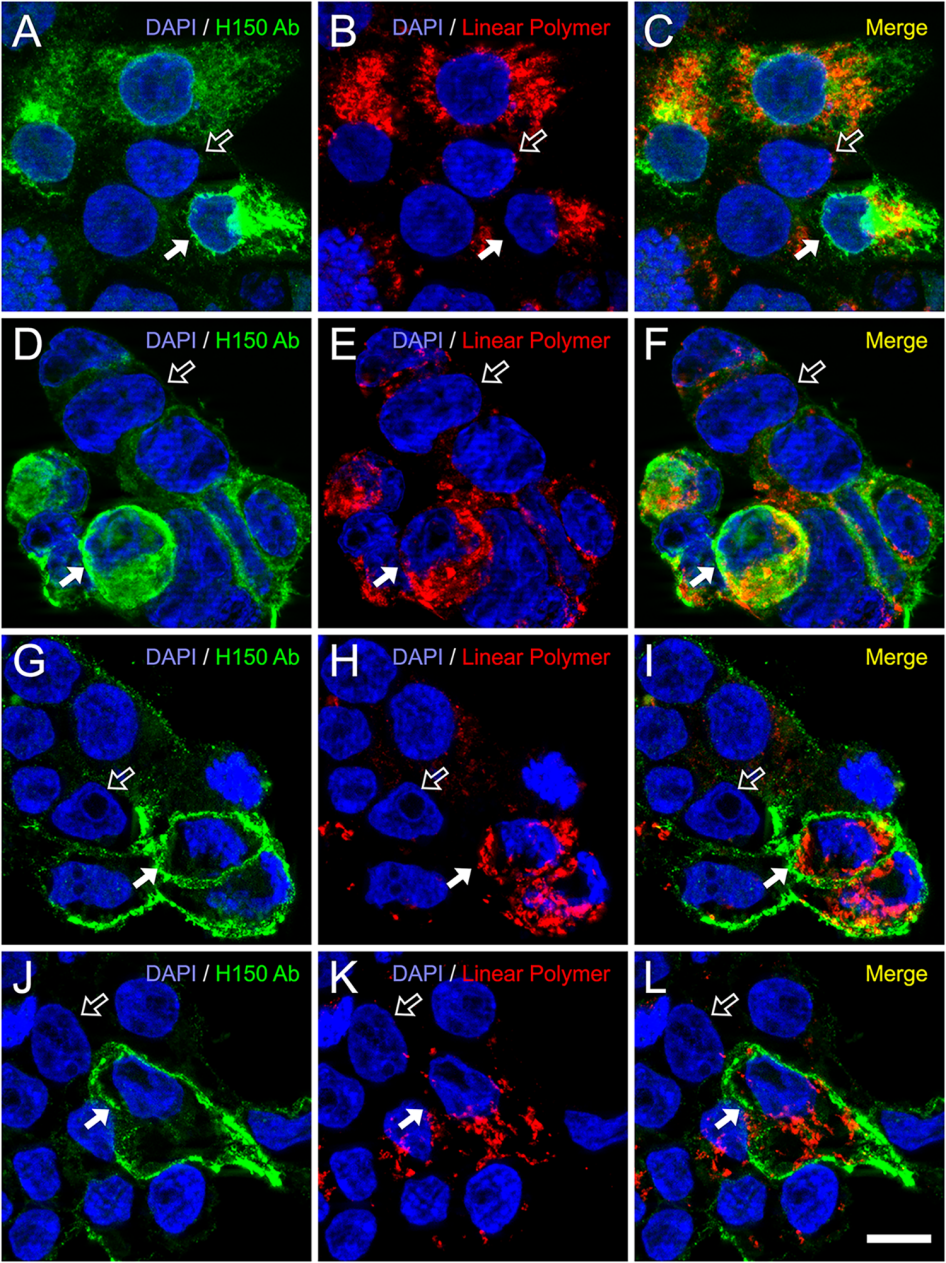
Double fluorescence labeling of HEK293T cells transiently expressing the CB1 receptor using an anti-CB1 antibody and the biotinylated linear polymer fabricated here. Cells were colabeled with a rabbit polyclonal antibody targeting the extracellular N-terminal domain of the human CB_1_ receptor and a biotinylated linear polymer specific for the last 12 amino acids of its intracellular C-terminal tail. Detection was performed using Alexa Fluor 488-conjugated antirabbit IgG (green) and Alexa Fluor 549-conjugated streptavidin (red). Cell nuclei were counterstained with Hoechst 33342 (blue). (A–F). Cells were fixed and permeabilized prior to simultaneous incubation with both the antibody and the linear polymer, allowing access to both surface and intracellular epitopes. (G–L). Cells were first incubated live with the N-terminal antibody, then fixed and permeabilized before incubation with the linear polymer. This approach restricted antibody binding to surface-exposed domains while permitting the polymer to access both compartments. CB_1_ receptor-overexpressing cells (solid arrows) displayed strong signals for both probes, whereas nontransfected cells (open arrows) showed low or negligible labeling with the biotinylated linear polymer, supporting its specificity for the CB_1_ receptor C-terminal region. Images correspond to maximum intensity projections of six consecutive optical sections (0.24 μm apart), acquired using structured illumination (ApoTome) on a Carl Zeiss Axio Observer microscope equipped with a motorized XYZ stage. Scale bar = 10 μm (applies to A–L).

In both conditions, cells expressing high levels of CB_1_, as evidenced by strong CB_1_ N-terminal immunoreactivity, also displayed robust red signal corresponding to polymer binding (Figure 5; solid arrows), whereas adjacent nontransfected cells (Figure 5; open arrows) exhibited negligible or very faint red fluorescence. This differential pattern supports the specificity of the polymer for the CB_1_ receptor and, more precisely, for its C-terminal domain. Despite the clear overlap in cell populations labeled by both reagents, a complete spatial colocalization was not observed, particularly in the surface compartment. This partial mismatch may reflect biological constraints that differentially affect epitope accessibility. While antibody binding to the N-terminal region is likely unaffected by intracellular interactions, the polymer target epitope resides within the cytoplasmic tail and may be influenced by protein–protein interactions, post-translational modifications, or conformational masking that hinder polymer binding. Such factors could underlie the imperfect colocalization, particularly in the complex plasma membrane milieu, where dynamic interactions with signaling proteins are expected.^50^ When the concentration of the biotinylated polymer was reduced to 10^–8^ M, signal discrimination between CB_1_ receptor-expressing and nonexpressing cells was almost lost (data not shown), likely due to a drop in signal-to-background ratio. This effect may reflect an increased contribution of off-target binding from the fluorophore-conjugated streptavidin used to detect the polymer, which becomes prominent at lower polymer concentrations and in the absence of saturating levels of specific interaction. These results validate the use of linear polymers in dual-labeling immunofluorescence experiments and confirm their capacity to recognize CB_1_ receptor-expressing cells, while also underscoring the influence of epitope accessibility and detection sensitivity in determining spatial signal distribution.

## Conclusions

Herein, the benefits of controlled PI-RAFT polymerization have been exploited for the production of fit-for-purpose linear polymers to be used as artificial receptors targeting the cannabinoid CB1 receptor. This work has served to demonstrate that the synthesis of linear polymers on the solid phase, guided by the presence of a short flexible peptide on that solid phase, induces the formation of a polymer that later works as a receptor, binding selectively to that peptide ligand. To reach this conclusion, the flexible intracellular C-terminus of the CB1 receptor was selected as the model epitope, for which a flexible linear polymer was produced, achieving outstanding recognition capacities with affinities comparable to those of natural antibodies. Blue light-mediated PI-RAFT polymerization has proven to be essential for this purpose, given that it has enabled the controlled synthesis of linear polymers of different particular sizes, well characterized by different chromatographic techniques. All produced polymers were explored as possible candidates as artificial receptors for the recombinant CB_1_ protein, concluding that the longest sizes provided the highest affinities. The polymers with the highest affinity were exploited for the recognition of the human CB_1_ receptor in cells expressing such protein by dual-labeling immunofluorescence. The extracellular N-terminal and intracellular C-terminal regions were successfully colabeled with an N-terminal antibody and the linear polymer (2×), respectively, which demonstrates the viability of these experiments using linear polymers as antibody mimics. The results derived from this work reveal that linear polymers produced here constitute a versatile alternative to natural receptors and might find wide application as antibody substitutes in different bioassays.

## Supplementary Material



## Notes

Additional experimental section: instruments; glass bead functionalization and covalent linkage of the peptide selected as target; results and discussion: adaptation of PI-RAFT polymerization to solid-phase synthesis in the presence of glass beads; optimization of SPR conditions; SPR evaluation of binding affinities of linear polymer using gold sensor slides having the C-Ter peptide attached; supporting figures: AlphaFold-predicted 3D structure of the human CB_1_ receptor (UniProt P21554) (Figure S1); photoreactor assembly (Figure S2); *M*_n_ and polydispersity indices (*M*_w_/*M*_n_) as a function of conversion (Figure S3); monomer conversion and pseudo-first-order kinetic plots (Figure S4); relationship between molecular weight evolution and conversion (Figure S5); overtime evolution of the *M*_n_ for linear polymers synthesized in the presence of increasing masses of peptide attached GB (Figure S6); schematic representation of the (3-iodopropyl)­trimethoxysilane (IPTMS) and (3-aminopropyl)­triethoxysilane (APTES) protocols (Figure S7); overtime evolution of the *M*_n_ for linear polymers without any iodine blocking, or after iodine blocking with mercaptoethanol (Figure S8); overtime evolution of *M*_n_ for linear polymers synthesized with different photoreactors (Figure S9); ^1^H NMR stacked spectra in D_2_O of (a) the RAFT agent, (b) HEEA, (c) 3-APMA, (d) NIPAm, (e) the control polymer, and (f) the linear polymer synthesized in the presence of the peptid**e** (Figure S10); ^1^H NMR spectra of (a) the control polymer and (b) the target linear polymer (Figure S11); optimization of working conditions for SPR experiments (Figure S12); fitted SPR sensograms on a gold sensor having the target C-Ter peptide attached (Figure S13); and observed relative angle shift (Δθ) decreases in the competition experiment (Figure S14) (PDF)
