# Supplementary material for: Controlled Photoiniferter RAFT-Based Development of Linear Polymers Induced by the Presence of a Peptide to Produce Synthetic Antibody Substitutes Applicable to Immunofluorescence Imaging Techniques
Source: Anal Chem. 2025 Dec 8;98(1):619–32. doi: 10.1021/acs.analchem.5c05598 (PMC12809648; doi:10.1021/acs.analchem.5c05598)
Supplement: Supplementary file 1 [file ac5c05598_si_001.pdf]

Supporting Information

Controlled Photoiniferter RAFT-Based Development of Linear Polymers Induced by the Presence of a Peptide to Produce Synthetic Antibody Substitutes Applicable to Immunofluorescence Imaging Techniques

Lucía Diez-Caballero[1], Imanol González-Burguera[2,5], Miquel Saumell-Esnaola[3,5], Nora Unceta[1,4], M. Aránzazu Goicolea[1,4], Joan Sallés[3,5], Ramón J. Barrio[1,4], Gontzal García del Caño[2,5], Alberto Gómez-Caballero[1,4].*

[1]Departments of Analytical Chemistry, [2]Neurosciences, and [3]Pharmacology, Faculty of Pharmacy, University of the Basque Country UPV/EHU, 01006 Vitoria-Gasteiz, Spain.

[4]Bioaraba, MetaboloMIPs, 01008 Vitoria-Gasteiz, Spain.

[5]Bioaraba, Neurofarmacología Celular y Molecular, 01008 Vitoria-Gasteiz, Spain.

*Corresponding author: a.gomez@ehu.eus

Table of Contents

EXPERIMENTAL SECTION 3

Instruments. 3

Glass bead functionalization and covalent linkage of the peptide selected as target. 4

RESULTS AND DISCUSSION 5

Adaptation of PI-RAFT polymerization to solid-phase synthesis in the presence of glass beads. 5

Optimization of SPR conditions. 8

SPR evaluation of binding affinities of linear polymer using gold sensor slides having the C-Ter peptide attached. 9

SUPPORTING FIGURES 11

Figure S1. 11

Figure S2. 11

Figure S3. 12

Figure S4. 13

Figure S5. 14

Figure S6. 15

Figure S7. 15

Figure S8. 16

Figure S9. 16

Figure S10. 17

Figure S11. 18

Figure S12. 19

Figure S13. 19

Figure S14. 20

REFERENCES 20

EXPERIMENTAL SECTION

Instruments. The molecular weight of synthesized polymers was monitored using an Agilent 1200 series chromatograph consisting of an isocratic pump, a column thermostat and an autosampler. The system was coupled to a 1260 Infinity series refractive index detector (RID) and multi detector suite with a multi-angle (15º and 90º) light scattering detector (LS) (Agilent Technologies). Agilent GPC/SEC Software was used for system control and data analysis. Monomer conversion was determined using an Agilent 1260 Infinity series chromatograph provided with a binary pump and an autosampler (injection volume 20 μL). The system was coupled to a 1290 Infinity II Diode Array Detector (DAD) working at a wavelength of 230 nm (Agilent Technologies). For system control and data analysis, the software Agilent OpenLab was used.

^{1}H nuclear magnetic resonance (NMR) spectra were recorded on a Bruker Advance 400 equipped with iProbe SmartProbe™. The substrates were dissolved in D_2O and transferred to a 5-mm NMR tube. NMR experiments were recorded at 298 K. Chemical shifts (δ) in ppm are referenced to tetramethylsilane (TMS) at 0.00 ppm. Coupling constants (J) are reported in hertz. The pulse conditions were as follows for all tested compounds: frequency (SF) = 400.13 MHz, spectral width (SW) = 8196.721 Hz, acquisition time (AQ) = 3.997 s, relaxation delay (RD) = 2.0 s, pulse width = 12.85 μs, flip angle = 30 º, number of scans = 64 (APMA, NIPAm, HEAA and the RAFT agent), 256 (control polymer), 8192 (target linear polymer). ***N*-(3-Aminopropyl)methacrylamide (APMA).** ^{1}H NMR (400 MHz, D_2O) δ 5.72 (broad s, 1H, $=CH_2$), 5.48 (broad s, 1H, $=CH_2$), 3.38 (broad s, 2H, CH_2N, 3.03 (broad s, 2H, CH_2N), 1.94 (broad s, 5H, CH_2 + CH_3) ppm (broadening of the peaks may be due to slow rotation in D_2O. ***N*-Isopropylacrylamide (NIPAM).** ^{1}H NMR (400 MHz, D_2O) δ 6.24-6.12 (m, 2H, =CH + $=CH_2$), 5.71 (dd, $^3J_{HH}$ = 9.7 Hz, $^2J_{HH}$ = 2.2 Hz, 1H, $=CH_2$), 3.96 (hept, $^3J_{HH}$ = 6.6 Hz, 1H), 1.15 (d, $^3J_{HH}$ = 6.6 Hz, 6H, 2×CH_3). **(2-Hydroxyethyl)acrylamide (HEAA).** ^{1}H NMR (400 MHz, D_2O) δ 6.24 (dd, $^3J_{HH}$ = 17.1 Hz, $^3J_{HH}$ = 9.9 Hz, 1H, =CH), 6.15 (dd, $^3J_{HH}$ = 17.2 Hz, $^2J_{HH}$ =1.8 Hz, 1H, $=CH_2$), 5.72 (dd, $^3J_{HH}$ = 9.9 Hz, $^2J_{HH}$ =1.8 Hz, 1H, $=CH_2$), 3.64 (t, $^3J_{HH}$ = 5.6 Hz, 2H, CH_2N), 3.36 (t, $^3J_{HH}$ = 5.6 Hz, 2H, CH_2N). **4-[[(2-**

Carboxyethyl)thio]thioxomethyl]thio]-4-cyanopentanoic acid (RAFT agent). ^{1}H NMR (400 MHz, D_2O) δ 3.65 (t, $^3J_{HH}$ = 7.0 Hz, 2H, CH_2S), 2.86 (t, $^3J_{HH}$ = 7.0 Hz, 2H, CH_2CO), 2.67 (t, $^3J_{HH}$ = 7.4, 2H, =CH_2CO), 2.20-2.62 (m, 2H, CH_2), 1.90 (s, 3H, CH_3).

SPR measurements were conducted using the MP-SPR Navi 210A VASA Multi-Parametric SPR instrument from BioNavis, having two independent fluidic channels in contact with the surface of the gold sensor, a PEEK temperature-controlled dual channel flow-cell, and an elastomer-coated prism. SPR measurements were conducted at 670 nm, in angular scan mode (55-75º), a scan time of 3.85s, and a temperature of 37ºC. Obtained SPR sensograms were processed by the software Data Viewer (Bionavis), and fitted using TraceDrawer analysis software (Ridgeview Instruments). The dual channel flow cell allows for immobilizing ligands just in one channel, while using the other as reference. Here, the target ligand was immobilized on the gold surface section where the fluid of channel 1 flows (primary channel), whereas the gold surface section of channel 2 was used as reference. SPR sensograms recorded on channel 1 refer to total binding, and signals recorded on channel 2 happen as a result to non-specific binding of the analyte and the gold surface, which was modified with the same coupling reagents as channel 1 but without having attached the target ligand. Gold sensor slides (Bionavis) were used for all SPR experiments.

Fluorescence imaging was conducted on an Axio Observer.Z1 epifluorescence microscope (Carl Zeiss) equipped with a HXP 120 C light source and a 63× Plan-Apochromat oil objective (NA 1.4). Images were acquired using an AxioCam MRm camera and processed via the ApoTome module and motorized XYZ stage, with a final pixel size of 0.01 μm^2. Filters used were: DAPI (Ex 365/Em 445/50), eGFP (Ex 470/40, Em 525/50), and Cy3 shift-free (Ex 550/25, Em 605/70). Acquisition was done with AxioVision 4.8; minor despeckling with ImageJ; and figures assembled in Adobe Photoshop 26.8.1 (Adobe Systems).

Glass bead functionalization and covalent linkage of the peptide selected as target. Linear polymers produced in this work were synthesized by the solid-phase approach, on the surface of glass beads (GB) of 150-210 µm in diameter (Merck) used as solid support, where the target peptide was

covalently attached prior to polymerization. For this, GB were activated in a boiling 1 M NaOH solution for 30 min, then thoroughly washed with water and acetone, and dried in an oven (100ºC, 2h). Next, GB were silanised using two approaches, first, using the aminosilane (3-aminopropyl)triethoxysilane (APTES) (approach 1), and, second, using an iodosilane such as (3-iodopropyl) trimethoxysilane (IPTMS) (approach 2). For approach 1, activated GB (120 g) were immersed in a 3% APTES solution prepared in a 95:5 ethanol:water mixture containing acetic acid (1 mL). This mixture was heated to 70 ºC for 1h, and then it was removed from the heat source and maintained at room temperature (RT) for another 24h. Next, GB-APTES were separated by filtration, washed with water and acetone, and cured in an oven (150 °C, 1.5h). Dry GB (20 g) bearing NH2 groups were immersed for 1.5h in a succinimidyl iodoacetate (SIA) solution (1 mg mL-1) prepared in phosphate buffer (0.1 M, pH 7.4), and then washed with ultrapure water. Finally, GB-APTES-SIA were added to a solution of the peptide (0.5 mg mL^{-1}) in borate buffer (0.1 M, pH 8.3), which contains an additional cysteine residue in its sequence (C-MSVSTDTSAEAL) to favor oriented coupling through the –SH group of cysteine, thereby having the peptide always attached in the same orientation. This mixture was left to react for 1.5h, and then, GB were thoroughly washed with ultrapure water.

For approach 2, after GB activation in NaOH, they were immersed in a 2% IPTMS solution in toluene for 24h. Then, they were washed with acetone, dried under vacuum and placed in an oven (150 °C, 1.5h). Since IPTMS itself provides pendant iodine groups to the surface of GB, there was no need to attach the short cross-linker SIA. Therefore, GB-IPTMS (20 g) were directly added to the peptide solution (0.5 mg mL^{-1}) and the same steps were followed as for approach 1.

RESULTS AND DISCUSSION

Adaptation of PI-RAFT polymerization to solid-phase synthesis in the presence of glass beads. After establishing the conditions that supported proper PI-RAFT based synthesis of linear polymers in solution, it was examined if polymer growth also proceeded properly in the presence of

glass beads having the C-Ter peptide attached. With this purpose, for a total monomer concentration of 0.6 M, a series of linear polymers were synthesized in 25 mL of 0.05 M phosphate buffer (pH 7.4) in the presence of 7.5, 10, 15, 20 and 30 g of peptide-immobilized GB, and the obtained polymers were analyzed by GFC-RID/LS to determine *Mn* evolution over time. As shown in Figure S6, all tested amounts of GB influenced polymerization to a greater or lesser extent, and 30 g drastically disrupted the process. Based on these findings, amounts not exceeding 20 g may be recommended for PI-RAFT based solid-phase synthesis of linear polymers, as the influence of this mass was assumable, causing an *Mn* decrease of about 15%.

Peptide grafting to the surface is a preliminary step prior to polymer synthesis, and it plays a major role in this work, since it influences the solid-phase synthesis of linear polymers conducted thereafter. Thus, proper immobilization of the target peptide by means of a covalent bond, which must be stable enough throughout the entire synthesis process, was essential. As detailed in the Methods section, two approaches have been explored for peptide immobilization. One of them is based on GB silanization using the silane (3-aminopropyl)triethoxysilane (APTES), whereas the other uses (3-iodopropyl)trimethoxysilane (IPTMS), which has proven to be a good alternative to amino silanes for solid-phase synthesis of imprinted materials.[1] APTES silanization introduces amino groups on the GB surface with (GB-NH_2), which subsequently react with the short heterobifunctional cross-linker succinimidyl iodoacetate (SIA), giving rise to terminal iodines (GB-NH-SIA). These iodines react with sulfhydryl groups of the target peptide at basic pH, resulting in a stable thioether linkage (Figure S7). Since the target peptide did not bear a native –SH group, it was purchased with an additional cysteine (**C**-MSVSTDTSAEAL) for oriented immobilization onto functionalized GB, thereby having it always attached from N- to C-terminus on the solid support. The iodosilane approach was initially selected for further experiments due to its greater simplicity, and also to reduce the total amount of reagents used for peptide immobilization. Nevertheless, this decision led us to encounter several experimental obstacles later on. We observed that using iodosilanes as substrates for peptide attachment had a negative influence on PI-RAFT polymerization. To reach this

conclusion, linear polymers were synthesized in the presence of 20 g of GB having the peptide attached via either the IPTMS or the APTES protocol (Figure S8), monitoring *Mn* evolution over 4h in both cases. When IPTMS was used obtained linear polymers were considerably shorter (965 g mol^{-1} *versus* 1704 g mol^{-1} after 4h), which suggested slower polymer propagation. This may be due to the greater number of free unreacted iodine groups present on the GB surface following peptide attachment, compared to the APTES-SIA protocol. which act as degenerative transfer agents in iodine-mediated reversible-deactivation radical polymerization, being crucial for controlling the polymerization process.[2–4] To demonstrate this, an additional synthesis was conducted in the presence of GB after peptide attachment by the IPTMS protocol, in which unreacted iodines were blocked with mercaptoethanol. As it can be deduced from Figure S8, *Mn* of polymers increased considerably when iodines were blocked before polymer synthesis, achieving sizes close to those obtained with the APTES protocol. In conclusion, residual iodine blocking was found to be crucial, not only because they interfere with the PI-RAFT process, but also to avoid polymer growth that may start from alkyl iodines on the GB surface. In any case, the APTES protocol was preferred here, to avoid the excessive number iodines introduced by IPTMS, and because it provided a higher yield of peptide grafting, achieving 27.7 ± 4.6 µg of bound peptide per g of GB, *versus* 14.0 ± 1.6 µg g^{-1} for GB-ITPMS determined by the Pierce BCA protein assay kit (Fisher Scientific).

^{1}H NMR analysis of polymers. NMR analysis of the linear polymer were performed for further characterizing the fabricated material. As reference, a control polymer was synthesized employing the same composition as the target linear polymer, but in the absence of any GB or peptide in the polymerization solution. These experiments served to determine whether the resulting target or control polymers contained the same type of monomer units. This information could help us better understand the extent to which the presence of the peptide in solution influenced the monomer composition of the polymer. The ^{1}H NMR spectrum of the polymer generated in solution (Figure S10e) reveals characteristic peaks that correspond to its four structural components. The presence of

the hydrophobic monomer NIPAm in the polymer structure is evident from the appearance of a broad signal at δ =3.88 ppm, which corresponds to the CH unit of the isopropyl group. The broad peak observed at δ =3.38 ppm is assigned to the methylene group, adjacent to the amino moiety of the basic monomer APMA. This signal appears to overlap with the one corresponding to the methylene group of the RAFT agent. Additionally, the presence of the uncharged polar monomer HEAA in the polymer structure is deduced from the broad peak at δ = 3.66 ppm, which correspond to the methylene group adjacent to the alcohol. Although almost imperceptible, the small broad multiplet at 2.59 ppm corresponds to the diastereotopic protons of the methylene group adjacent to the chiral carbon in the structure of the RAFT compound, which also confirms its presence in the final structure of the polymer. Similarly, the ^{1}H NMR spectrum of the target linear polymer synthesized in the presence of the C-Ter peptide (Figure S10f) shows the characteristic peaks corresponding to the four components of its structural components with slight deviations in their chemical shifts, and substantial changes in the integrations if compared with the polymer generated in solution. According to the integration of the most characteristic signals of the four components of the control polymer synthesized in solution (Figure S11a), the estimated composition was 43.7% NIPAm, 34.2% HEAA, 18.0% APMA and 4.1% RAFT agent. In contrast, the composition of the target linear polymer produced in the presence of the peptide (Figure S11b) was 33% NIPAm, 44.3% HEAA, 17.8% APMA and 4.9% RAFT agent.

Optimization of SPR conditions. Before conducting binding experiments, experimental conditions such as working temperature, the addition of NaCl and injection flow rate were optimized to maximize the total-to-non-specific signal ratio, registered as resonance angle shifts (Δθ) in flow-channels 1 and 2, respectively. These experiments were conducted using a gold sensor having attached the C-Ter peptide, and injecting a fixed concentration of the ×2 linear polymer (0.5 μmol L^{-1}) in the SPR system. As illustrated in Figure S12, the ratio was higher when working at 37ºC and in the absence of NaCl, which negatively affected binding. The parameter that contributed the most to specific binding was injection flow rate, which was studied between 5 and 40 μL min^{-1}. Total

binding (channel 1) increased considerably at low flow rates, whereas non-specific binding (channel 2) remained fairly similar. It was therefore deduced that peptide-polymer binding kinetics might be rather slow, requiring more time than usual for proper adaptation of the peptide and the linear polymer chains. However, operating continuously at the minimum flow rate allowed by the instrument (5 µL min^{-1}) was technically challenging, since it promoted air entry, bubble formation, and baseline drift. Accordingly, 10 µL min^{-1} was selected.

SPR evaluation of binding affinities of linear polymer using gold sensor slides having the C-Ter peptide attached. Different sensograms were recorded injecting increasing concentration of ×2 and ×1 polymers in the SPR system (Figure S13), which were subsequently fitted to the Langmuir one-site (1:1) binding model. Figure S13a and b depict fitted channel 1 (ch.1) sensograms after reference signal substraction from channel 2 (ch.2), to remove the contribution of non-specific binding of injected polymers to ligand-free areas on the gold surface, thus allowing the isolation of the signal attributable to polymer-peptide interactions. As it can be observed in the figure, angle shift signals for each tested concentration were higher for 2× polymers than 1×, which denoted higher binding. Furthermore, the dissociation-rate constant (k_d), which defines the stability of the formed ligand-polymer complex, was considerably lower for 2× polymers (5.20 ×10^{-5} s^{-1}), indicating the formation of a more stable complex than with 1× polymers, which exhibited a k_d of 21.2 ×10^{-5} s^{-1}. On the other hand, the association-rate constant was five times higher for 2× polymers (5.72 ×10^{3} M^{-1} s^{-1}) than for 1× polymers (1.17 ×10^{3} M^{-1} s^{-1}). Influenced by their lower k_d and higher k_a, 2× polymers showed a lower equilibrium dissociation constant (K_D) than 1× polymers (9.09 ± 0.27 nM and 181 ± 15 nM, respectively) reflecting higher affinity (Table 1).

To explore whether polymer-peptide binding was influenced or not by the linear arrangement of amino acids in the C-Ter peptide, while keeping its overall composition unchanged, polymer interaction with a scrambled version of the C-Ter peptide (LSMDEVSTATSA) was also examined. To this end, a gold sensor with the scrambled peptide was installed in the system, and increasing

concentrations of 2× (Figure S13c) and 1× (Figure S13d) linear polymers were injected. Depicted sensograms show angle shifts signals (Δθ) of ch.1 after substraction of ch. 2 signals. No signal to concentration dependence was observed for 2× or 1× polymers, and, moreover, experimental data did not fit to the one-site binding model. Therefore, it was deduced that polymer binding to the scrambled peptide was probably weaker than for the C-Ter peptide, and very likely driven by non-specific interaction with isolated amino acids or short motifs.

SUPPORTING FIGURES

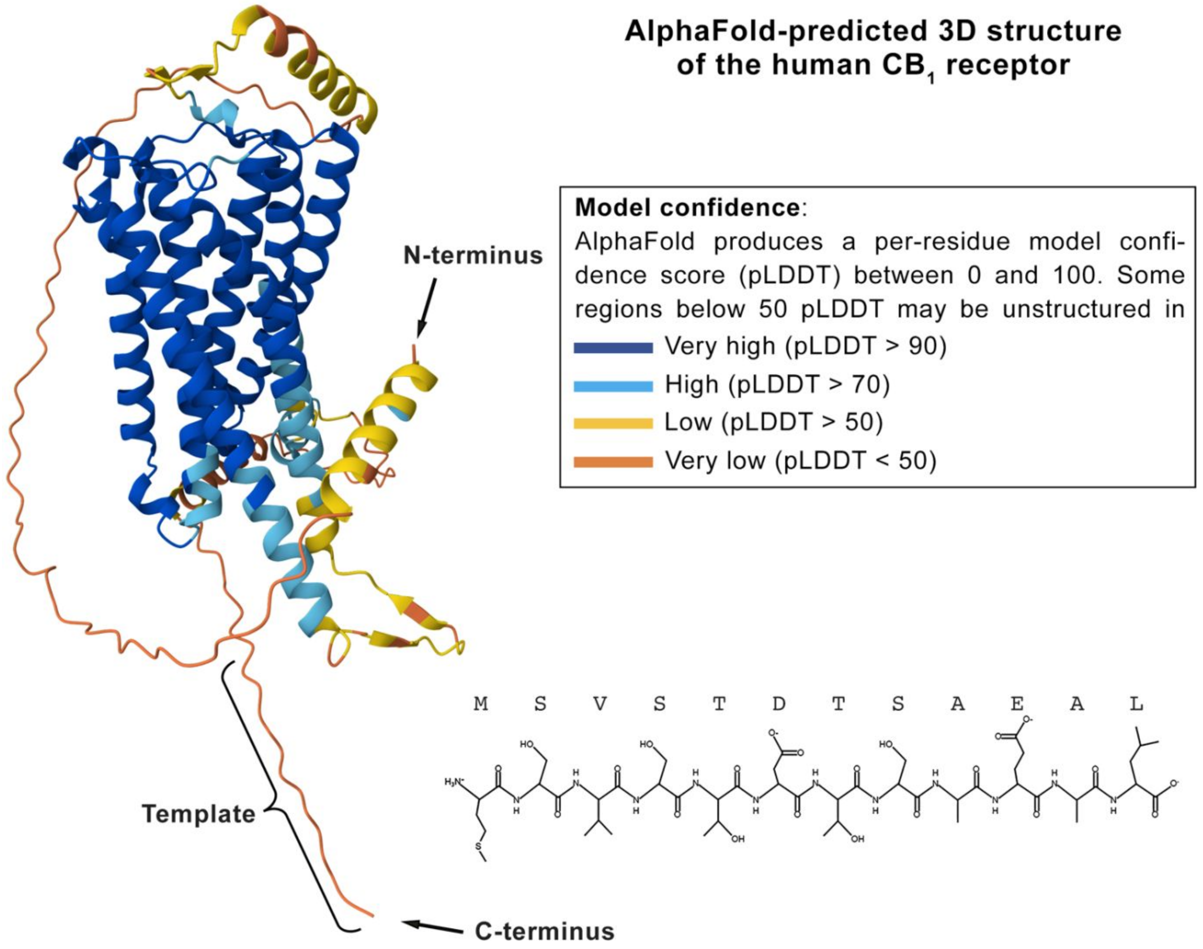

Figure S1. AlphaFold-predicted 3D structure of the human CB_1 receptor (UniProt P21554). The structure is coloured by per-residue model confidence (inset). The long cytosolic C-terminal tail shows low pLDDT. The distal segment used as the imprinting template (residues 461–472) is indicated and its 12-residue sequence (MSVSTDTSAEAL) is shown below as a linear chemical formula.

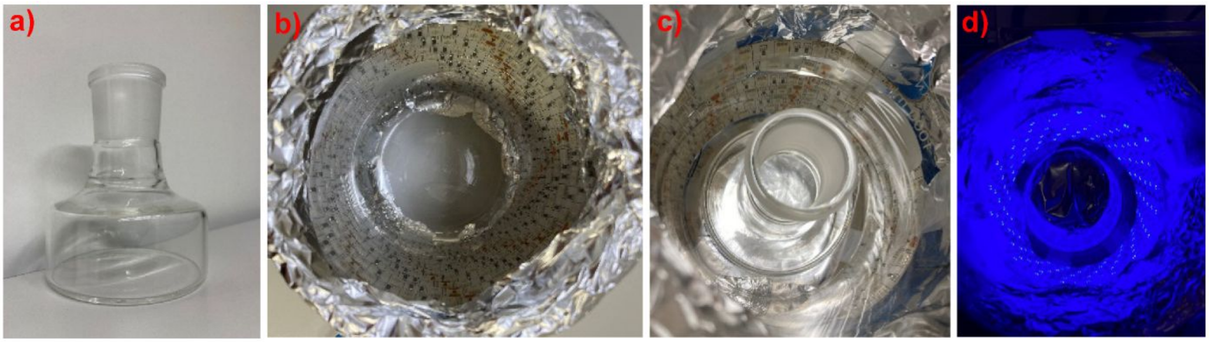

Figure S2. Photoreactor assembly including a) the custom-fabricated flask to work in an inert atmosphere, b) the arrangement of the LED strip around the 1L beaker, c) the placement of the flask inside the photoreactor, and d) the photochemical polymerization reaction irradiating the mixture with blue light.

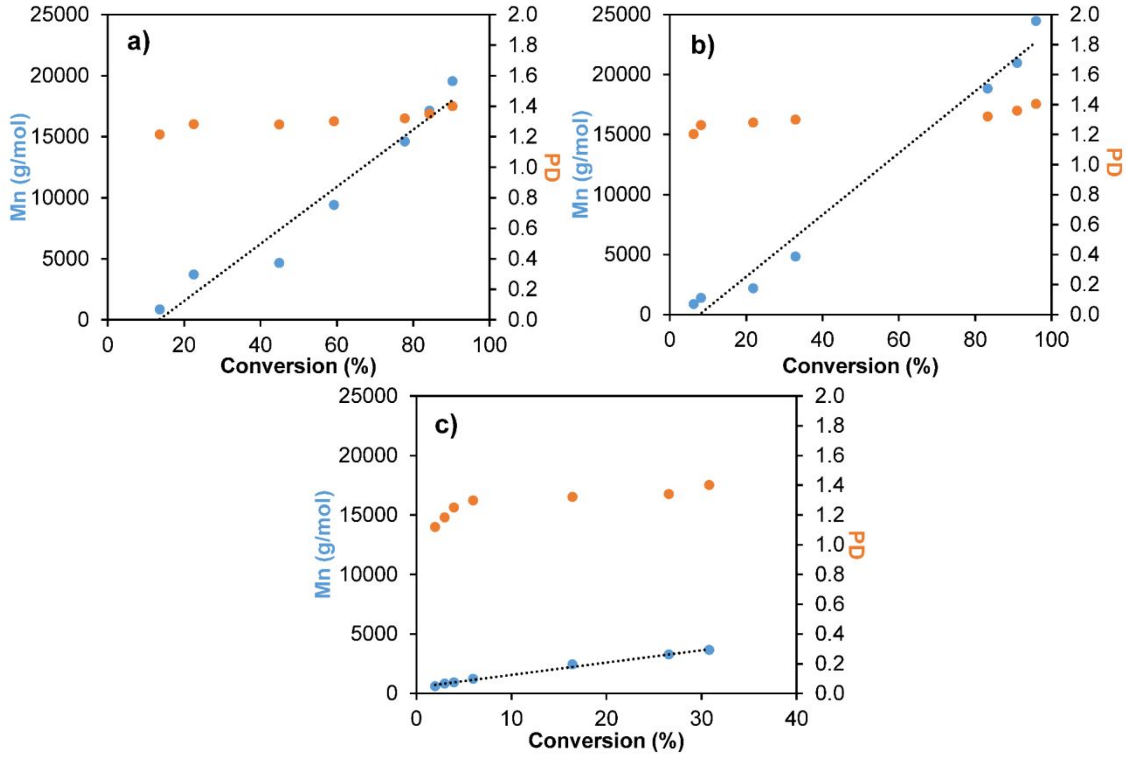

Figure S3. Mn and polydispersity indices (Mw/Mn) as a function of conversion for polymerization mixtures containing total monomer concentrations of a) 1.2 M, b) 0.6 M and c) 0.3 M. A [500]:[1] Monomer:RAFT ratio was used for all experiments.

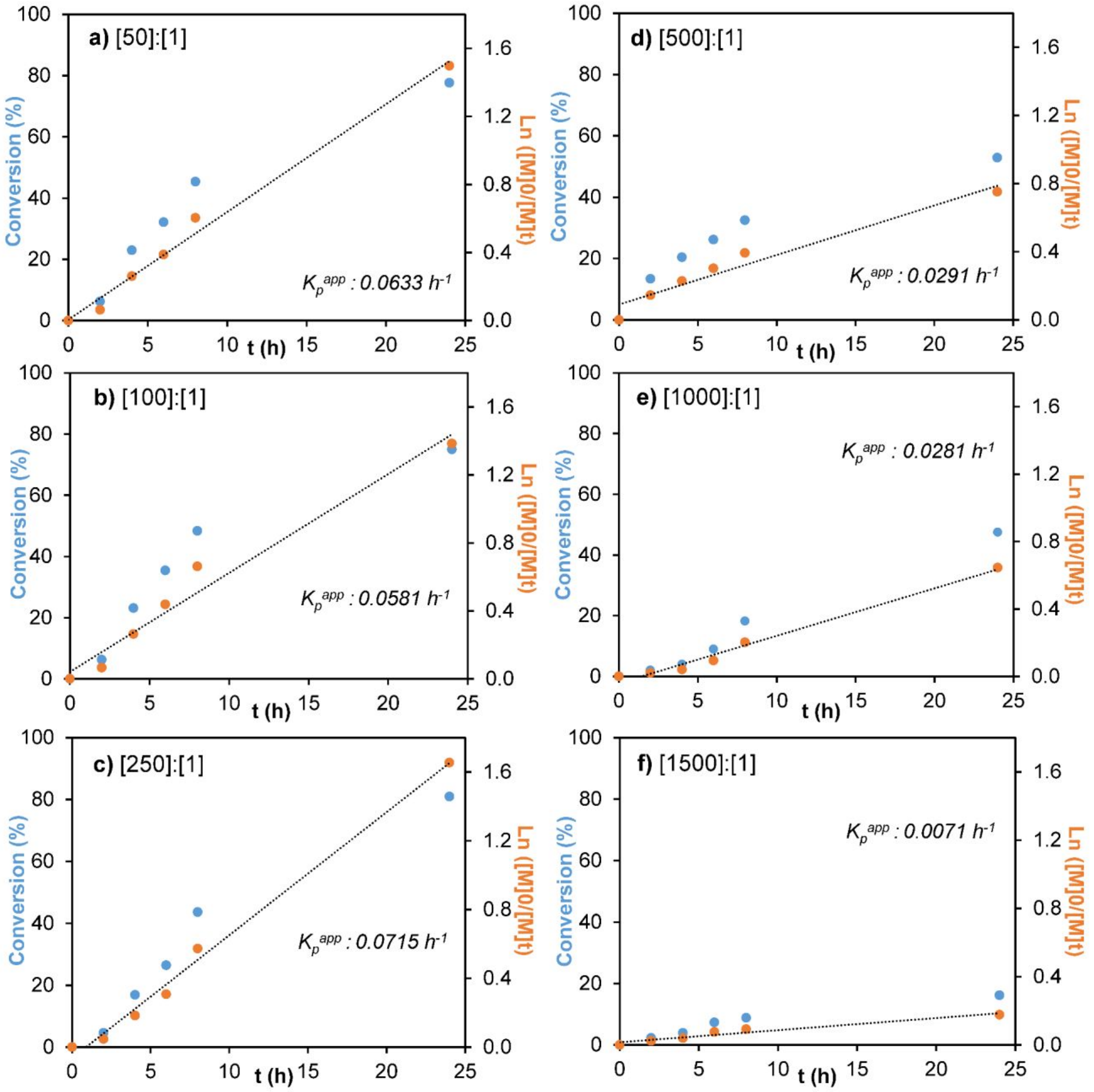

Figure S4. Monomer conversion (blue) and pseudo first kinetic plots (orange) for polymerization reactions carried out with mixtures having a total monomer concentration of 0.6 M and increasing [monomer]:[RAFT] molar ratios: a) [50]:[1], b) [100]:[1], c) [250]:[1], d) [500]:[1], e) [1000]:[1], and f) [1500]:[1].

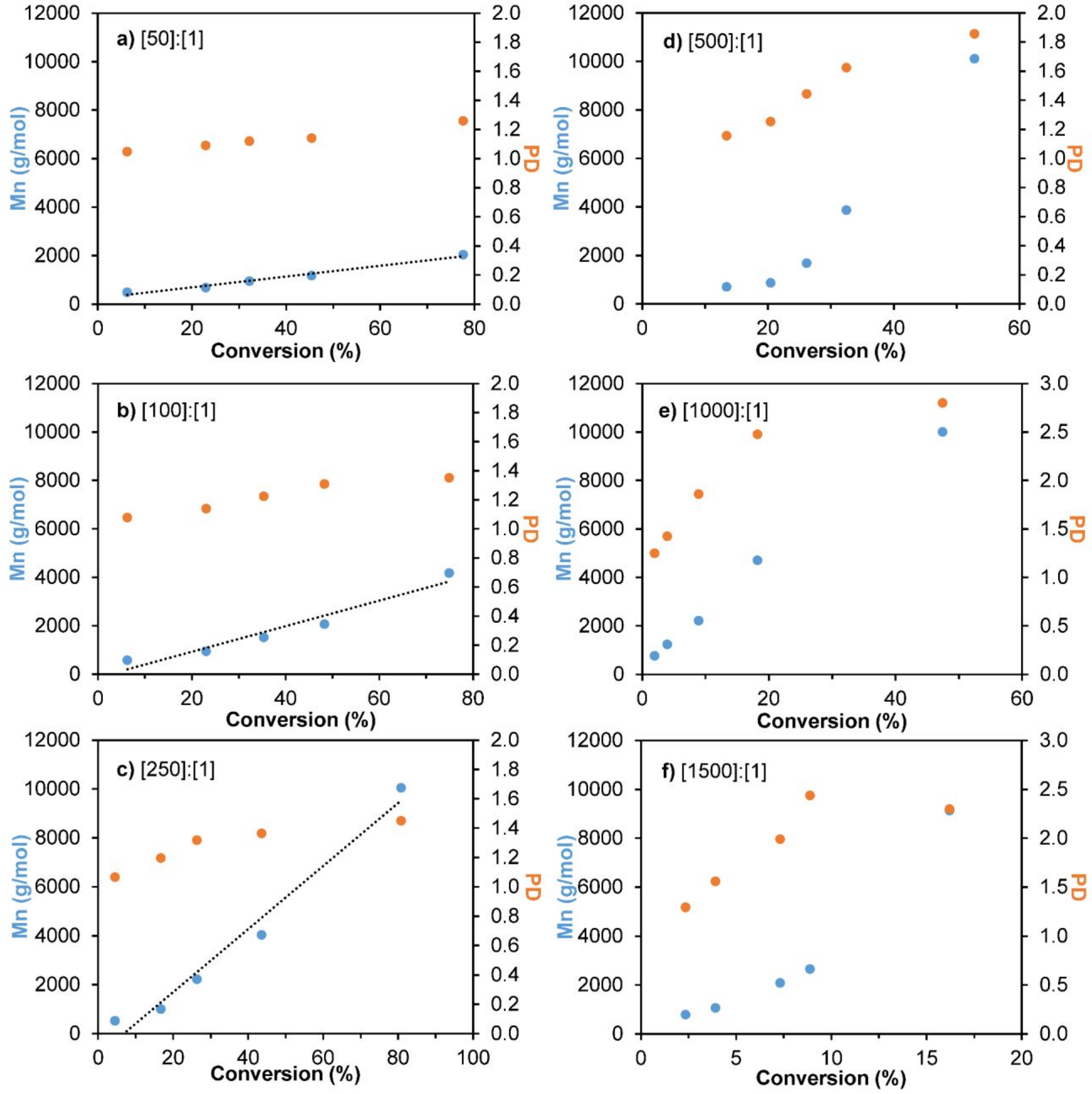

Figure S5. Depiction of the relationship between molecular weight evolution and conversion for polymerization reactions carried out with mixtures having a total monomer concentration of 0.6 M and increasing [monomer]:[RAFT] molar ratios: a) [50]:[1], b) [100]:[1], c) [250]:[1], d) [500]:[1], e) [1000]:[1], and f) [1500]:[1].

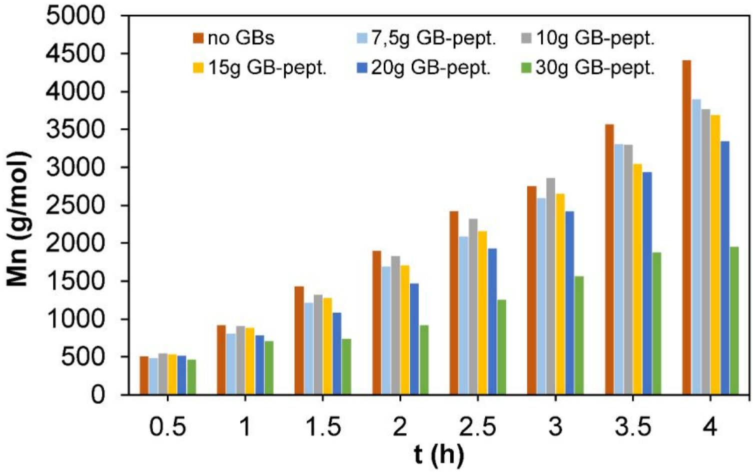

Figure S6. Over-time evolution of the Mn for linear polymers synthesized in the presence of increasing masses of peptide-attached GB. A 25 mL mixture containing a total monomer concentration of 0.6 M and a [250]:[1] [monomer]:[RAFT] ratio was used.

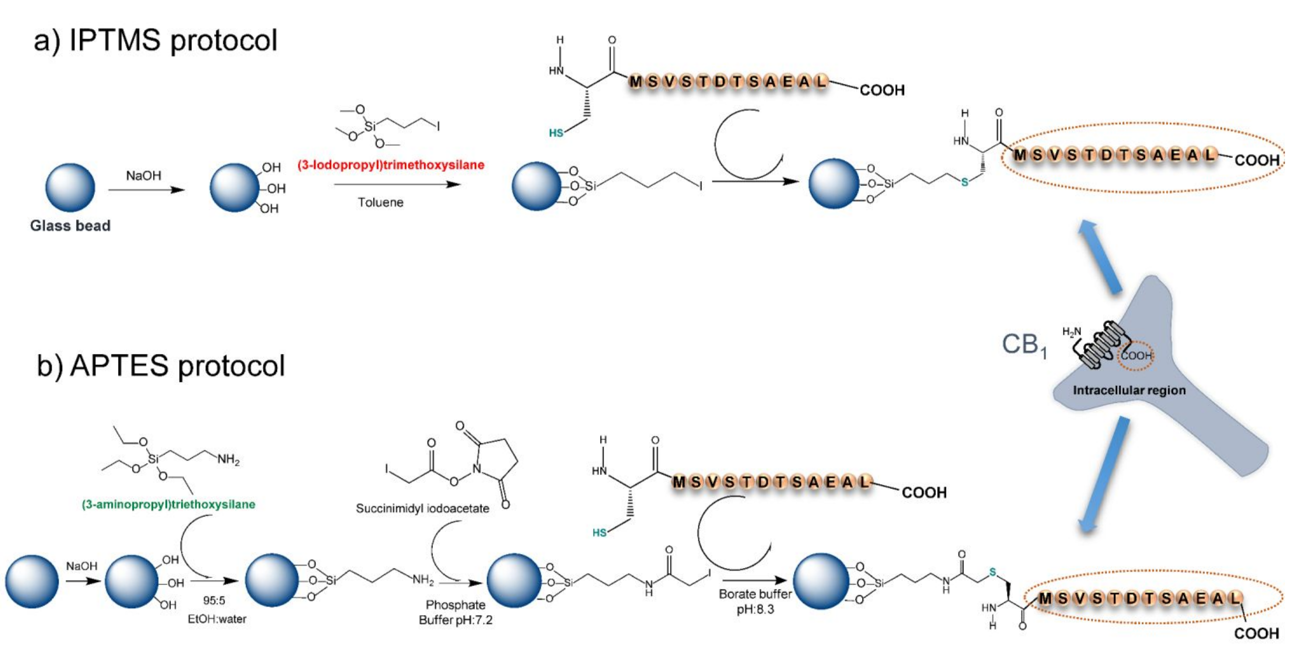

Figure S7. Schematic representation of the a) (3-iodopropyl)trimethoxysilane (IPTMS) and b) (3-aminopropyl)triethoxysilane (APTES) protocols followed to immobilize the target peptide on the surface of glass beads.

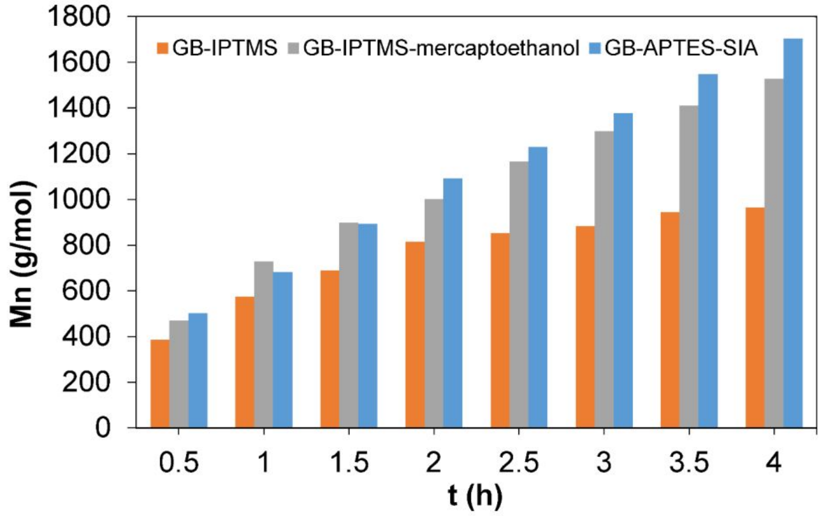

Figure S8. Over-time evolution of the Mn for linear polymers synthesized in the presence of 20 g of GB functionalized with the silanes APTES (blue bar) and IPTMS (orange bar), without any iodine blocking, or after iodine blocking with mercaptoethanol.

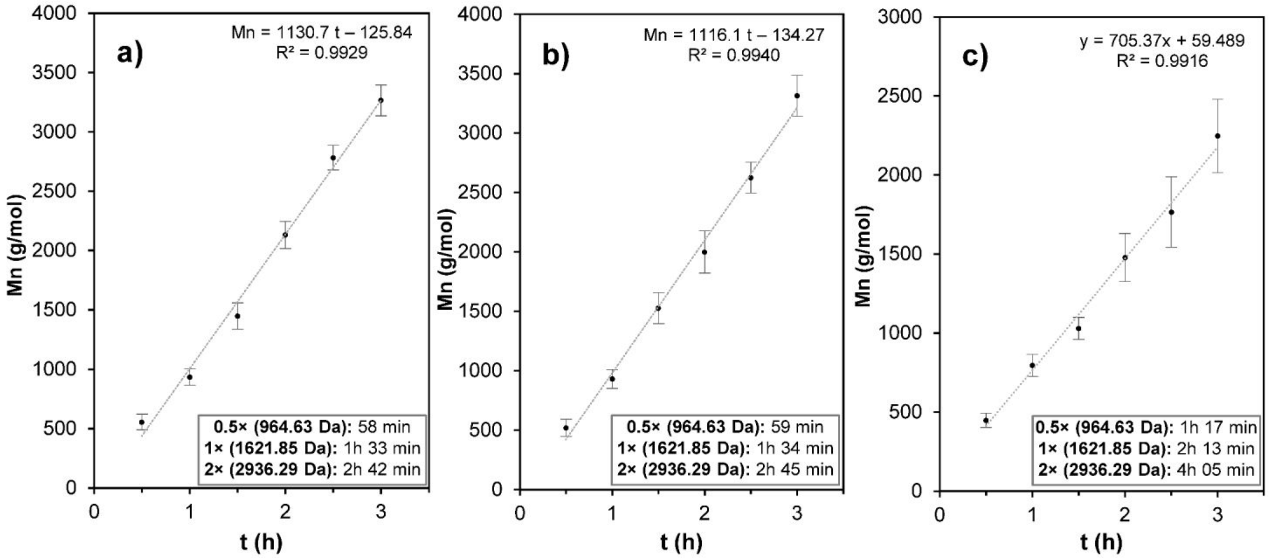

Figure S9. Over time evolution of Mn for linear polymers synthesized with different a), b) and c) photoreactors. A total monomer concentration of 0.6 M and a monomer:RAFT ratio of 250:1 was used in all cases. Inset boxes show irradiation times required to 2×, 1×, and 0.5× linear polymers.

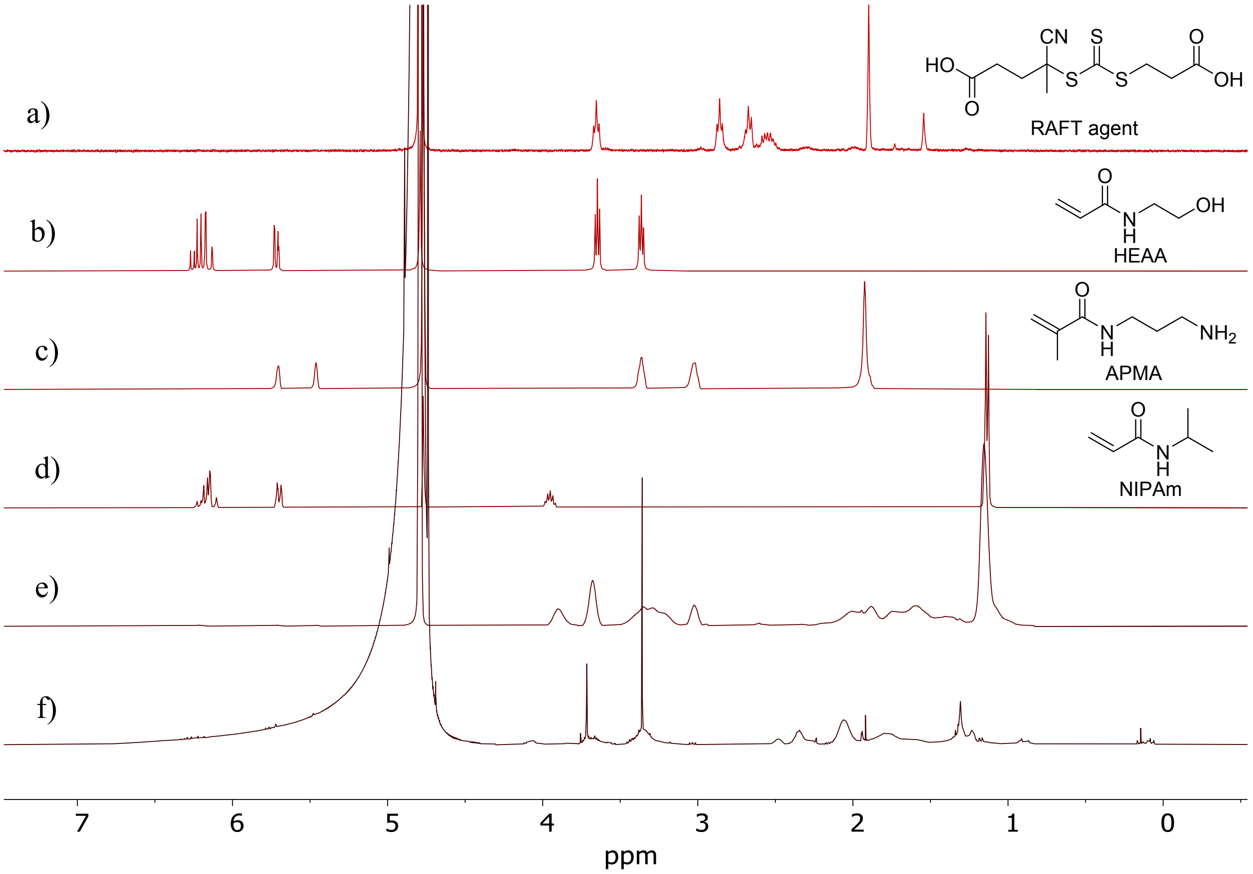

Figure S10. ^{1}H NMR stacked spectra in D_2O of (a) the RAFT agent, (b) HEEA, (c) APMA, (d) NIPAm, (e) the control polymer, and (f) the linear polymer synthesized in the presence of the peptide.

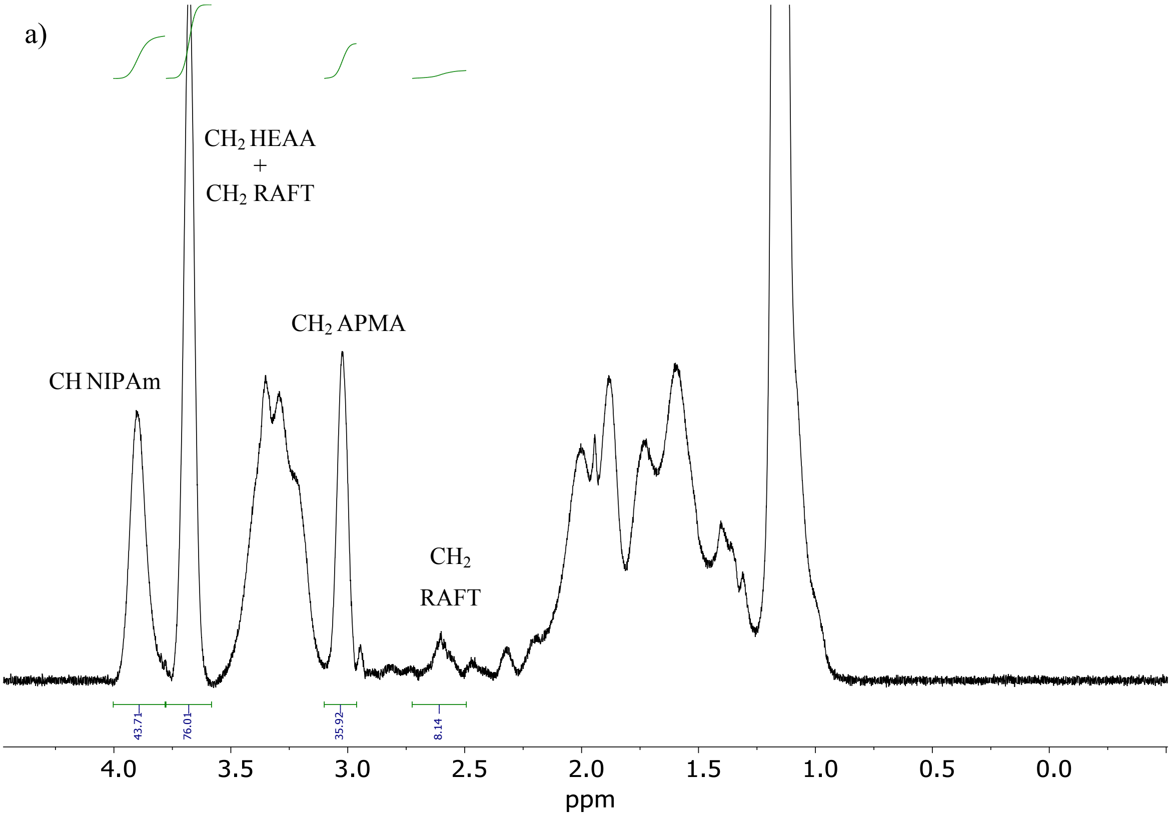

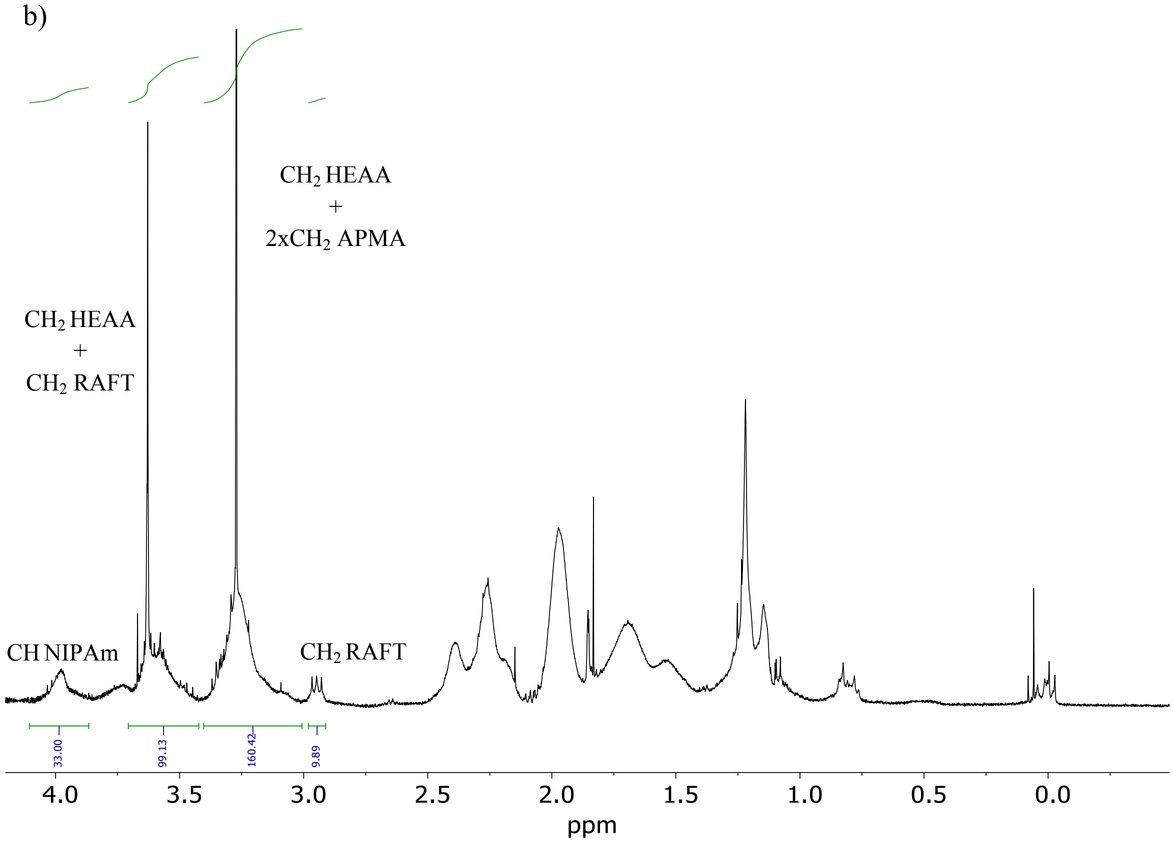

Figure S11. ^{1}H NMRspectra of a) the control polymer, and b) the target linear polymer.

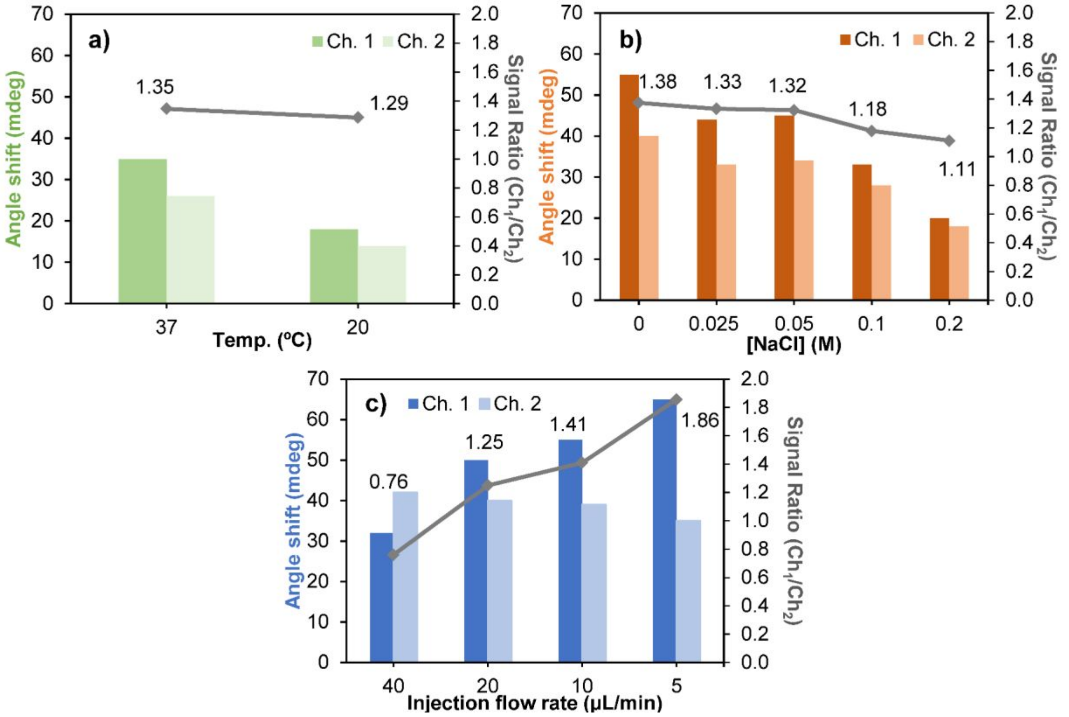

Figure S12. Optimization of working conditions for SPR experiments. Influence of the a) working temperature, b) NaCl concentration in the running buffer, and c) injection flow rate on maximum angle shift signals recorded in channels 1 (primary channel) and 2 (reference channel).

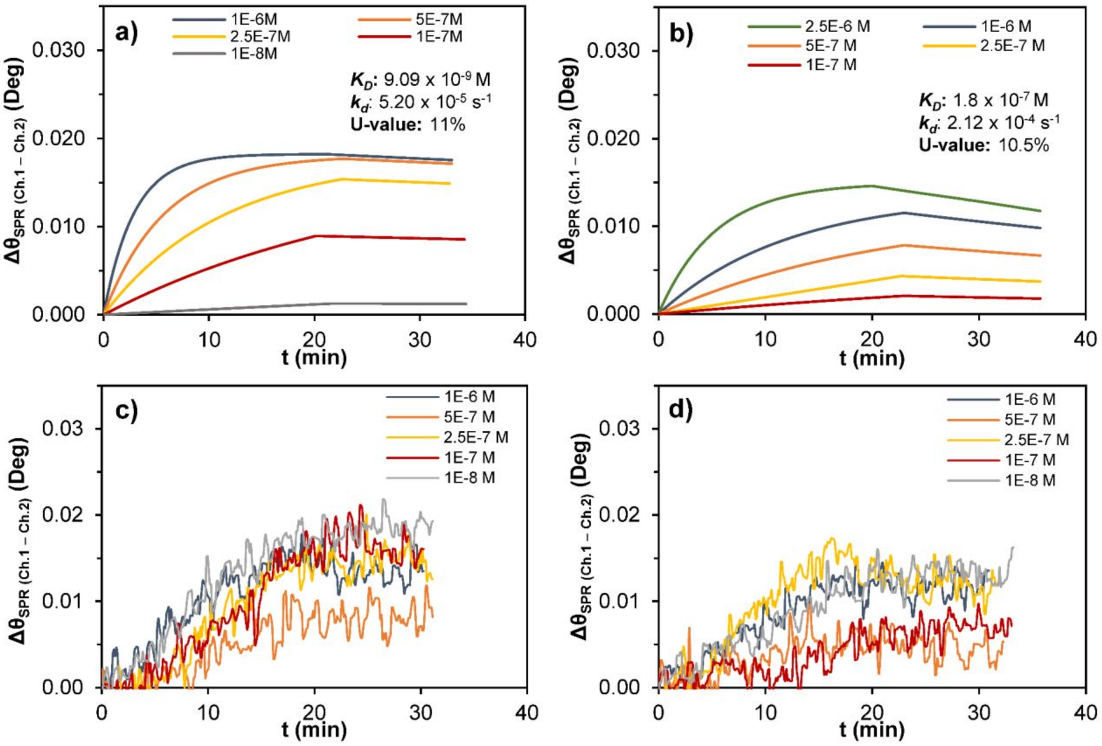

Figure S13. Fitted SPR sensograms injecting increasing levels of a) 2× and b) 1× linear polymers on a gold sensor having the target C-Ter peptide attached. Whereas SPR sensograms for increasing concentrations of x1 and x2 linear polymers are shown in figures c) and d) respectively, using as ligand the scrambled version of the C-Ter peptide attached to the gold sensor.

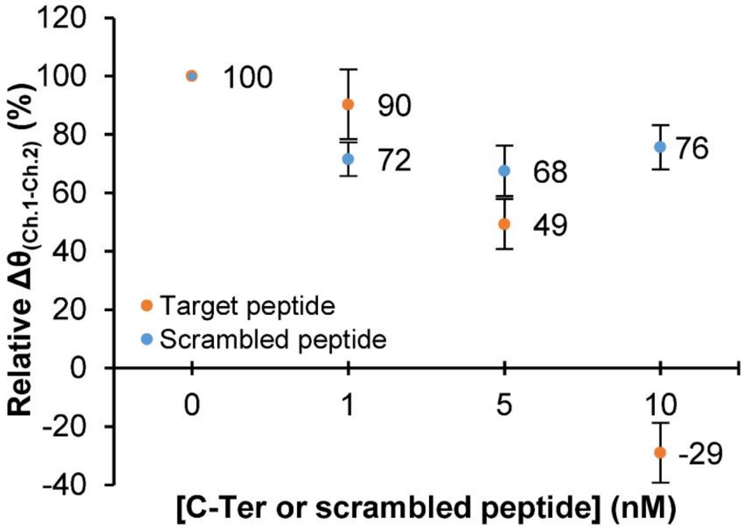

Figure S14. Observed relative angle shift (Δθ) decreases for 100 nM of the GST-CTer protein after pre-injecting depicted concentrations of the target peptide (orange dots) or the scrambled peptide (blue dots).

REFERENCES

(1) Piletsky, S. S.; Garcia Cruz, A.; Piletska, E.; Piletsky, S. A.; Aboagye, E. O.; Spivey, A. C. Iodo Silanes as Superior Substrates for the Solid Phase Synthesis of Molecularly Imprinted Polymer Nanoparticles. *Polymers (Basel)*. **2022**, *14* (8), 1595. https://doi.org/10.3390/polym14081595.

(2) Ni, Y.; Zhang, L.; Cheng, Z.; Zhu, X. Iodine-Mediated Reversible-Deactivation Radical Polymerization: A Powerful Strategy for Polymer Synthesis. *Polym. Chem.* **2019**, *10* (20), 2504–2515. https://doi.org/10.1039/c9py00091g.

(3) Kumru, B.; Antonietti, M. Emerging Concepts in Iodine Transfer Polymerization. *Macromol. Chem. Phys.* **2023**, *224* (3), 1–6. https://doi.org/10.1002/macp.202200316.

(4) Ni, Y.; Tian, C.; Zhang, L.; Cheng, Z.; Zhu, X. Photocontrolled Iodine-Mediated Green Reversible-Deactivation Radical Polymerization of Methacrylates: Effect of Water in the Polymerization System. *ACS Macro Lett.* **2019**, *8* (11), 1419–1425. https://doi.org/10.1021/acsmacrolett.9b00507.
